# Parsing the Role of the Hippocampus in Approach–Avoidance Conflict

**DOI:** 10.1093/cercor/bhw378

**Published:** 2016-12-18

**Authors:** Eleanor Loh, Zeb Kurth-Nelson, David Berron, Peter Dayan, Emrah Duzel, Ray Dolan, Marc Guitart-Masip

**Affiliations:** 1 Wellcome Trust Centre for Neuroimaging, University College London, London WC1n 3BG, UK; 2 Max Planck UCL Centre for Computational Psychiatry and Ageing Research, University College London, London WC1B 5EH, UK; 3 Institute of Cognitive Neurology and Dementia Research, Otto-von-Guericke University, D-39120 Magdeburg, Germany; 4 Gatsby Computational Neuroscience Unit, University College London, London W1T 4JG, UK; 5 Institute of Cognitive Neuroscience, University College London, London WC1N 3AR, UK; 6 Ageing Research Centre, Karolinska Institute Stockholm, SE-11330 Stockholm, Sweden

**Keywords:** anxiety, approach–avoidance, exploration, decision-making, hippocampus

## Abstract

The hippocampus plays a central role in the approach–avoidance conflict that is central to the genesis of anxiety. However, its exact functional contribution has yet to be identified. We designed a novel gambling task that generated approach–avoidance conflict while controlling for spatial processing. We fit subjects’ behavior using a model that quantified the subjective values of choice options, and recorded neural signals using functional magnetic resonance imaging (fMRI). Distinct functional signals were observed in anterior hippocampus, with inferior hippocampus selectively recruited when subjects rejected a gamble, to a degree that covaried with individual differences in anxiety. The superior anterior hippocampus, in contrast, uniquely demonstrated value signals that were potentiated in the context of approach–avoidance conflict. These results implicate the anterior hippocampus in behavioral avoidance and choice monitoring, in a manner relevant to understanding its role in anxiety. Our findings highlight interactions between subregions of the hippocampus as an important focus for future study.

## Introduction

Approach–avoidance conflict arises when animals encounter probabilistic gains and losses within the same experience and are thus forced to balance the desire to seek reward with the impulse to avoid harm. Such conflict between approach and avoidance is thought to be central to the generation of anxiety, a state of high arousal and negative valence that is experienced in the absence of an immediate threat (Russell 1980; Davis and Shi 1999; Gray and McNaughton 2000; Calhoon and Tye 2015). Behavior in approach–avoidance situations as commonly used in rodent anxiety assays is highly sensitive to both anxiolytic drugs (Borsini et al. 2002) and hippocampal lesions (particularly to the anterior hippocampus, corresponding to the ventral hippocampus in rodents; Bannerman et al. 2003; Pentkowski et al. 2006; see Gray and McNaughton 2000 for review). More recently, evidence has emerged implicating the hippocampus in anxiety and approach–avoidance processing in humans as well (Bach et al. 2014; O'Neil et al. 2015).

Theoretical work suggests different ways in which the hippocampus may contribute to the processes underlying anxiety (Gray and McNaughton 2000). First, the hippocampus may monitor for conflict between impulses to approach and avoid. Second, it may inhibit ongoing behavior once conflict is detected. Third, it may initiate information seeking or risk-assessment activities to gather more information about alternative courses of action. To date, experimental attempts to disentangle these distinct contributions have been lacking. Hippocampal damage has been demonstrated to result in impulsivity and deficits in behavioral inhibition in the context of an approach–avoidance conflict (Abela et al. 2013; Bach et al. 2014). Evidence for a role of this structure in information seeking more generally (i.e., outside approach–avoidance situations) is mixed (Daw et al. 2006; Simon and Daw 2011; Badre et al. 2012; Bornstein and Daw 2012, 2013; Wang and Voss 2014). Results do suggest that the hippocampus is involved in forward planning in humans and vicarious trial-and-error behavior in rodents (which emerges when animals are unsure about which choice to make during learning; see Johnson et al. 2007 and Redish 2016 for review). However, although forward planning and information seeking may, to some extent, depend on shared neuronal mechanisms, it is unclear whether such hippocampal-dependent processes are more likely to be engaged specifically in the context of approach–avoidance conflict (as suggested by Gray et al.).

An additional unresolved issue relates to whether the hippocampus is specifically involved in approach–avoidance conflict, or whether instead it merely represents the episodic context within which such conflict occurs. This latter possibility is theoretically important given the role of the hippocampus in representing spatiotemporal contexts (e.g., as noted in Rudy et al. 2002; see Burgess et al. 2002 for review). Although links between anxiety and hippocampal anatomy have been noted in humans (Barrós-Loscertales et al. 2006; Levita et al. 2014), most functional studies of approach–avoidance conflict have hitherto employed experimental paradigms with significant spatiotemporal components (e.g., Bach et al. 2014, Hasler et al., 2007; though note a recent exception in O'Neil et al. 2015). As such, the extent to which the hippocampus’ contribution relates mostly to representing the spatiotemporal context in approach–avoidance situations remains unclear.

To address these issues, we employed functional magnetic resonance imaging (fMRI) alongside a novel approach–avoidance (Ap/Av) gambling task that enabled us to separate hippocampal contributions to avoidance and exploration. We controlled for nonspecific processes unrelated to aversion (e.g., memory, planning, spatial processing) by including an approach–approach (Ap/Ap) condition in which subjects faced the same probabilistic gambles without the threat of loss, and instead had to make choices according to what they thought was most likely to be rewarded. By allowing subjects to choose explicitly between rejecting aversive gambles and exploring them (i.e., risk assessment, requesting more information), we tested whether the hippocampal contributions to choice in an approach–avoidance context related to behavioral avoidance or to information gathering (exploration). A first model-agnostic analysis of choice-related signals revealed a segregation of signals within the anterior hippocampus: the inferior hippocampus (potentially overlapping with the CA1 subfield) distinguished between avoiding an instrumentally aversive gamble and acknowledging an abstract threat that posed no instrumental harm, whereas the superior hippocampus (potentially overlapping with the CA3 subfield) did not show this distinction. Next, we employed a model-based approach focusing on value signals that may have been calculated by subjects doing our task, and showed that superior hippocampal value signals are uniquely potentiated in the context of approach–avoidance conflict, consistent with a role in monitoring ongoing experiences for conditions that necessitate an avoidant response.

## Materials and Methods

### Subjects

Thirty-nine adults completed training sessions on the task, of whom 21 were selected for imaging (see Experimental Procedure for detail). Of these 21 subjects, 1 was excluded for poor MRI coverage. Thus, a total of 20 subjects completed all sessions of the experiment and were included in the analyses (9 male; mean age = 22.60 years, standard deviation [SD] = 2.39). All subjects were right-handed, had normal or corrected-to-normal vision, and none reported a history of neurological/psychiatric conditions. All subjects gave written informed consent, according to the local ethics clearance committee (No. 3793/001, University College London, UK). Anxiety scores were collected (State-Trait Anxiety Inventory [STAI]; Spielberger and Gorsuch 1970), and correlated with other data using Spearman's rank correlation test (nonparametric test used because the anxiety scores were not normally distributed; Kolmogorov–Smirnov test, *D*(20) = 0.22, *P* < 0.01). Note that although the use of STAI is thought to index individual differences in anxiety, this metric is not able to completely separate anxiety from depressive traits, given the high level of correlation between these traits in the general population (Kaneda and Fujii 2000; Bados et al. 2010; Hill et al. 2013).

### Experimental Task

**Figure 1.**
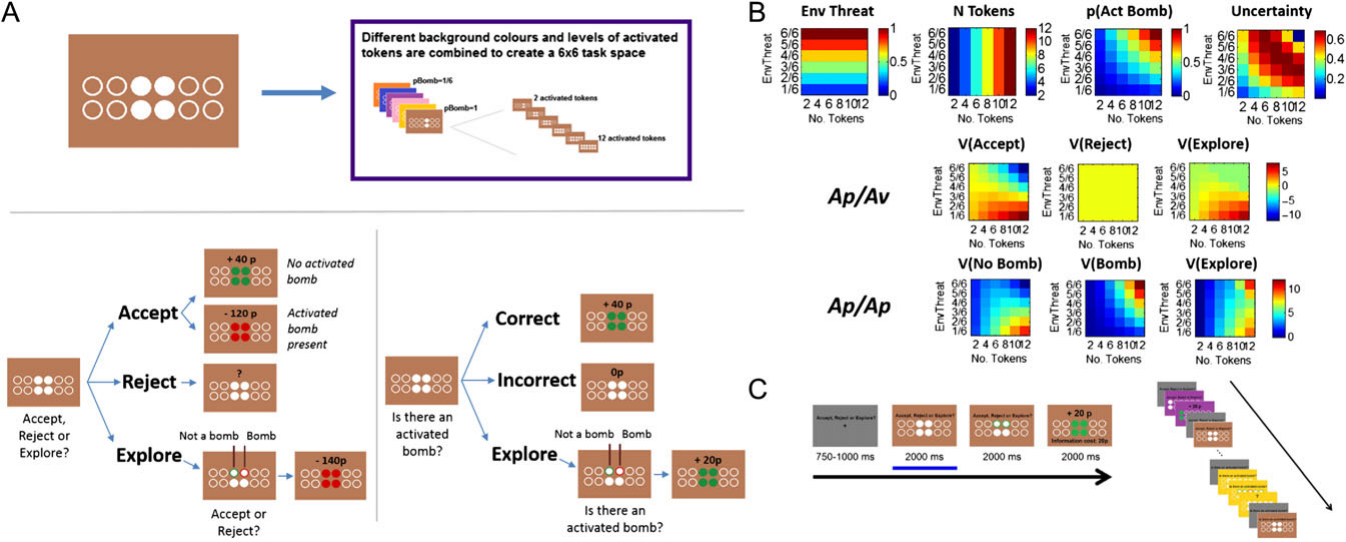
Experimental design. Subjects evaluated gambles (*A*, top) for the presence of an activated bomb. Gambles comprised different combinations of 6 background colors (environmental threat) and 6 levels of activated tokens, combined to create a 6 × 6 task space (*B*). Choice in the Ap/Av condition (accept, reject vs. explore) involved a risk of win or loss (*A*, bottom left), while choice in the control task (bomb, no bomb, explore) never resulted in loss (*A*, bottom right), and subjects were rewarded for correctly guessing if a bomb was present or not (see Results/Experimental Procedure for detail). In the scanner, subjects made choices in response to each gamble, with the condition (Ap/Av or Ap/Ap) clearly indicated onscreen (*C*). All fMRI results relate to the evaluation and choice period of the trial sequence (indicated by blue bar in *C*, left), which was decorrelated from the outcome-presentation and exploration stages by omitting these latter stages in 50% of all trials.

Our task manipulated the extent to which approach and avoidance impulses were pitted against each other, while controlling for other nonspecific psychological factors (i.e., similarly to existing paradigms like the Columbia Card Task, which does not however allow subjects the option to collect more information before proceeding; Figner et al. 2009). Subjects experienced 2 conditions (approach–avoidance, Ap/Av and approach–approach, Ap/Ap) with the same design, but different semantics and consequences for the actions (see Fig. 1*A*). On each trial, subjects saw a certain number of tokens (filled circles) on a colored background indicating potential rewards. Hidden among all the possible locations might be a bomb; but it would only be “activated” if it was planted under one of the tokens in the array. The different colored backgrounds indicated different probabilities that a bomb had been planted (ranging from 1/6 to 1; termed “environmental threat” and abbreviated “EnvThreat” in the figures). If it was planted, then it would be placed randomly at any of the 12 sites; so its probability of it exploding increased with the number of tokens presented on a trial (colored in white; i.e., Fig. 1*A* shows a gamble with 4 activated tokens), ranging from 1 to 6 pairs. Thus, the probability of an activated bomb (*P*(ActBomb)) is

$$\begin{array}{c} P\left(\mathrm{ActBomb}\right)=P(\mathrm{Bomb}\;\mathrm{planted})\times P(\mathrm{Bomb}\;\mathrm{activated}|\mathrm{planted})\\ =\mathrm{Env}\;\mathrm{Threat}\times \frac{No.\,\;of\;\,\mathrm{activated}\;\mathrm{tokens}}{12} \end{array}$$

A maximum of one bomb was planted in a gamble on any given trial. Over the course of the experiment, subjects faced different combinations of 6 environmental threats and 6 levels of activated tokens. The task space thus comprised a 6 × 6 factorial design (environmental threat, number of tokens), totaling 36 unique gambles.

In the Ap/Av condition, subjects had 3 choices: accept (i.e., attempt to gather the rewards), reject (i.e., avoid the gamble), or explore. Accepting a gamble that did not contain an activated bomb resulted in subjects winning money (10p/token). However, accepting a gamble that did contain an activated bomb led to a fixed loss of −120p. Because the number of activated tokens additionally signaled how much money was available to win on a given trial, increasing the number of activated tokens simultaneously increased both potential winnings and *P*(ActBomb)), given the same environmental threat. Rejecting a gamble effectively discarded the gamble without incurring either gain or loss. Exploring required paying a 20p fee to discover whether or not there was a bomb under 50% of the tokens (Fig. 1*A*, bottom left). If subjects chose to explore on a trial in which the bomb was under a token, then there was a 50% chance that they would find this during exploration. Subjects could only explore once per trial. After exploring, they then had to decide whether to accept or reject the gamble, which then had the same pecuniary consequences as at the first stage. This procedure allowed us to separate avoidance and exploratory risk assessment, something that previous studies have not attempted.

In the Ap/Ap condition, subjects faced the same gambles (i.e., the same combinations of environmental threat and number of activated tokens, indicating the same *P*(ActBomb). However, instead of choosing to accept or reject the gamble, subjects had to guess whether it contained an activated bomb or not (Fig. 1*A*, bottom right). Subjects won money (10p/token) for each correct guess and incurred neither gain nor loss for incorrect guesses. As in the Ap/Av condition, subjects had the opportunity to explore the gamble (incurring the same 20p fee) before making their decision. Therefore, the Ap/Ap task required subjects to represent the available information and compute *P*(ActBomb) in a similar manner to the Ap/Av condition, but without the possibility of loss and, therefore, without any attendant avoidance.

### Experimental Procedure

One day prior to scanning, subjects completed a learning session, a session of the Ap/Av condition and a session of the Ap/Ap condition. During the learning session, the game was explained, and subjects learned the environmental threat associated with each background color by observing trials in which they were forced to accept gambles on every trial. On those trials, the outcomes followed the Ap/Av condition, that is, subjects lost money if an activated bomb was present, and otherwise won money proportional to the number of tokens on the trial (see Supplementary materials for detail). At the end of this training session, subjects completed a 2-alternative-forced-choice task in which they chose between 2 of the background colors at a time, and gave explicit ratings of the probability of a bomb being planted for each background color. Subjects whose performance on the explicit ratings or the forced-choice task indicated that they had failed to learn the order of the background colors (i.e., each of their rankings in terms of environmental threat) were excluded from further participation in the task. Winnings from this learning session did not count toward subjects’ monetary rewards, to prevent subjects from adopting a risk-averse strategy for subsequent sessions. Subjects then completed a session of the Ap/Av condition, followed by a session of the Ap/Ap condition, in which they made choices (accept/choose no bomb, reject/choose bomb, and explore) and observed the outcome on every trial (see Supplementary materials for detail). Subjects also explicitly ranked the background colors, and completed the same 2-alternative-forced-choice task (i.e., choosing between the background colors) after each session. We excluded from further participation subjects who 1) changed their ranking of the background colors in terms of ascending environmental threat after either of the Ap/Av or Ap/Ap task session (as this indicated a change of mind regarding the background colors from the training session); 2) indicated either in their rankings or in their behavior that they had mixed up the order of the background colors with respect to the associated threat levels (see Supplementary Fig. 5*A*, top, for examples); 3) failed to explore entirely (see Supplementary Fig. 5*A*, bottom, for examples); 4) could not verbally explain how information from the environmental threat and number of activated tokens should be combined to estimate *P*(ActBomb), which had been explained to them before the start of the experiment. These strict screening procedures were employed in order to select for fMRI the subjects who had best learned the structure of the task. This ensures that scanned participants robustly show the behavior that we were interested to study and increases the statistical power of our design. Furthermore, this allowed us to ensure that the scanned subjects would have reasonably similar estimates of *P*(ActBomb) across the 6 × 6 task space, and so ensure that the psychological variables that were controlled for in our fMRI model were reasonably accurate (Fig. 1*B*; see later sections for more detail). Despite these strict exclusion criteria, available choice behavior for the excluded subjects in these training sessions was, on average, similar to that of the included subjects (Supplementary Fig. 5*B*). Indeed, analysis of the choice probabilities with a group (included vs. excluded) × condition × choice ANOVA found no significant effects related to the subject group (all *P *> 0.3), though a main effect of choice and condition × choice interaction were found for the reported results when the sample was restricted to the high performers. This implies that there is continuity between included and excluded subjects and that the results of our experiment are generalizable.

During the fMRI session (performed the day after initial training and screening), we collected fMRI data while subjects completed 12 alternating blocks of the Ap/Av and Ap/Ap condition with the starting condition counterbalanced across subjects (Fig. 1*C*). Subjects were told that the *P*(ActBomb) associated with each gamble was the same as in previous sessions, and that this last session was an opportunity for them to use what they had learned so far to maximize their winnings in the game. The overall amount of money paid to subjects was proportional to overall accumulative winnings in the game (i.e., all sessions excluding the initial learning session).

All fMRI results presented relate to the stage of the trial sequence in which subjects were first evaluating gambles and indicating their choices (indicated by the blue bar in Fig. 1*C*, left). Activation relating to this evaluation period was decorrelated with processing of the outcome and any explored information, by omitting the latter 2 stages during 50% of the fMRI trials and including nuisance regressors describing these latter 2 stages in our fMRI models. Subjects were told that incomplete “explore” trials would be completed after the scanning session, but no such postscanning session was actually conducted, and subjects’ winnings for these trials were calculated by assuming that second-stage choice would conform deterministically to the information revealed during exploration. Subjects were only informed about how winnings for these incomplete explore trials were calculated after all scanning was completed. The fMRI stage of the experiment consisted of 1296 trials (18 repetitions of the 36 Ap/Av and 36 Ap/Ap trials), and subjects were offered a break after every other block. Only behavioral data from this stage were analyzed.

### Overview of the Data Analysis

We first analyzed our behavioral and fMRI data using a model-agnostic approach that focused on choice in the different experimental conditions. We followed this with a model-based analysis that enabled us to examine latent quantities (notably, subjective value) that might be used to guide strategic choice in our task. Behavioral modeling was employed to calculate the subjective values underlying choice in the task.

### Characterization of Task Space with Respect to Psychological Learning-Related Variables

We characterized the 6 × 6 task space in terms of several task-related variables that might be tracked at a psychological or neural level across the 6 × 6 task space (Fig. 1*B*). These variables were as follows: environmental threat, number of activated tokens, *P*(ActBomb), the uncertainty or entropy associated with this probability (as defined by Strange et al. 2005) and EV of nonexploration, calculated in the Ap/Av condition as:

(1) EV=−12×P(ActBomb)+n×(1−P(ActBomb))

where *n* is the number of tokens. In the Ap/Ap condition, EV was

(2) $$\begin{array}{c} \text{EV}=\max\left(V\left(\mathrm{Bomb}\right),\;V\left(\mathrm{No Bomb}\right)\right) \end{array}$$

V(No Bomb)=(1-P(ActBomb))×n

V(Bomb)=P(ActBomb)×n

While EV in the Ap/Av condition reflects the value of accepting the gamble, EV in the Ap/Ap condition reflects the amount of money that subjects may win if they make an accurate choice in the task. All variables (other than EV) took the same values across the task space in the Ap/Av and Ap/Ap conditions. However, the interpretation of *P*(ActBomb) differed between the 2 tasks, indicating the probability of losing money in the Ap/Av but not in the Ap/Ap conditions. These task-related variables served as the starting point with which to construct the computational models. The maximal correlation between these variables was *r* = −0.86, between *P*(ActBomb) and EV.

### Behavioral Modeling

Only choices from the fMRI session (i.e., after complete learning) were modeled, and choice on each trial was assumed to be independent. The model space was defined by first calculating the true EVs of accepting/choosing no bomb, rejecting/choosing bomb and exploring, and then parameterizing 7 separate possible sources of suboptimal influence over these values (see Supplementary materials for detail). The potential sources of suboptimality were identified by considering both errors in optimal calculation (*j* parameter: mis-estimation of EnvThreat; *m* parameter: systematic miscalculation of the *P*(ActBomb) after one explores and fails to see a bomb), as well as descriptive psychological tendencies that could interfere with optimal performance (*f* parameter: loss aversion; *e* parameter: a tendency for overvalue exploration in some circumstances; *i* parameter: a tendency to act according to null information revealed during exploration, rather than integrating this information into a revised estimate of *P*(ActBomb)).

In the winning behavioral models, the values in the Ap/Av condition (quantified in terms of tokens) were calculated as follows:

(3) $$\begin{array}{c} V\left(Accept\right)=a\times f+(1-a)\times n \end{array}$$

$$\begin{array}{c} V(Reject)=0 \end{array}$$

$$\begin{array}{c} \begin{array}{c} V\left(Explore\right)=P\left(See\right)\times V\left(See\right)+P\left(No\;See\right)\times V\left(No\;See\right)\\ -2+u\times w\\ a={\mathrm{Env Threat}}^{j}\times n/12 \end{array}\\ P(See)=\frac{1}{2}a\\ V(See)=0\\ P(No\text{​}\;See)=1-\frac{1}{2}a\\ V(No\;See)=k^{m}\times f+\left(1-k^{m}\right)\times n\;\;\;\;\;\;\;\;\;\;\;\text{if}>0\\ =0\;\text{otherwise}\left(\mathrm{gamble}\;\mathrm{rejected}\right) \end{array}$$

where *a* is the subjective probability of an activated bomb, *f* is the perceived magnitude of the fixed loss (which objectively equals −12 tokens), *n* is the number of activated tokens on that trial, *j* is a power law distortion of environmental threat, “See” describes the state of having seen an activated bomb during exploration (and whose value is 0 in the Ap/Av condition because it is assumed that a subject would reject the gamble at the second stage), “No see” describes not having seen an activated bomb during exploration, *u* is uncertainty regarding the probability of an activated bomb, *w* is an exploration bonus that quantifies the impact that uncertainty has on *V*(Explore), *k* is the posterior probability of an activated bomb given that exploration does not reveal an activated bomb (calculated using Bayes rule), and *m* is a power law distortion of *k* (i.e., describing suboptimal calculation of the posterior probability). We allowed for distortion of the posterior probability *k* to allow for the possibility that subjects may incorrectly integrate information from exploration to form updated estimates of *P*(ActBomb), despite having been well trained on the base rates of *P*(ActBomb) from the start.

Values in the Ap/Ap task were as follows:

(4) V(Accept)=(1−a)×n

V(Reject)=a×n

$$\begin{array}{c} V\left(\mathrm{Explore}\right)=P\left(\mathrm{See}\right)\times V\left(\mathrm{See}\right)+P\left(\mathrm{No}\;\mathrm{See}\right)\times V\left(\mathrm{No}\;\mathrm{See}\right)\\ +e+u\times w \end{array}$$

$$a={\mathrm{Env}\mathrm{Threat}}^{j}\times \mathrm{No}\;of\mathrm{Tokens}/12$$

$$P\left(\mathrm{See}\right)=\frac{1}{2}a$$

V(See)=n

$$P\left(\mathrm{No}\;\mathrm{See}\right)=1-\frac{1}{2}a$$

$$V\left(\mathrm{No See}\right)=V_{\mathrm{Stage}2\mathrm{Accept}}\mathrm{if}V_{\mathrm{Stage}2\mathrm{No Bomb}}>V_{\mathrm{Stage}2\mathrm{Bomb}}$$

$$=V_{\mathrm{Stage}2\mathrm{Reject}}\text{otherwise}$$

$$V_{\mathrm{Stage}\;2\;\mathrm{Accept}}=\left(1-k^{m}\right)\times n+i$$

$$V_{\mathrm{Stage}\;2\;\mathrm{Reject}}=k^{m}\times n$$

where *V*(See) = *n* (assuming subjects correctly indicate “bomb” on such trials), the variable exploration bonus *w* here quantifies the effect of uncertainty (*u*) on *V*(Explore)*, i* describes a bonus to *V*_Stage 2 No Bomb_, reflecting a general tendency to choose no bomb in which exploration does not reveal a bomb (as opposed to optimally integrating the null information into an estimate of *P*(ActBomb)).

Values for each choice were then used to predict the probability of accepting/choosing no bomb, rejecting/choosing bomb, or exploring on each trial via a softmax function. Separate models were included for all possible combinations of all free parameters considered (see Supplementary materials for detail; all fitted parameter values shown in Supplementary Table 6). Parameter fitting was implemented separately for the Ap/Av and Ap/Ap conditions using a hierarchical type II Bayesian (random effects) procedure that used maximum likelihood to fit simple parameterized distributions for higher level statistics of the parameters (Huys et al. 2011). Models were compared using the integrated Bayesian information criterion (iBIC), in which small iBIC values indicate a model that fits the data better after penalizing for the number of parameters (to prevent over-fitting).

### fMRI Analysis

Data were acquired with a functional resolution of 3 mm isotropic (structural resolution: 1.3 mm isotropic), and preprocessed prior to full analysis (see Supplementary materials for detail). Three different general linear models (GLMs) were constructed, to analyze data from several different perspectives: 1) “categorical choice,” 2) “chosen and counterfactual value,” and 3) “choice × value difference.” All models focused on the 2000-ms epoch during which the gambles were presented onscreen, wherein subjects had to make their choices (i.e., indicated by the blue bar in Fig. 1*C*). Orthogonalization of parametric modulators was omitted in the design matrix for all models, so as to ensure that parameter estimates relating to the regressors and parametric modulators compete for variance (Andrade et al. 1999). The outcome-presentation and exploration stages were omitted during 50% of all trials, in order to enable us to decorrelate the gamble evaluation and choice stage from all other stages in our task design. These events were also included as nuisance regressors in all fMRI models.

The categorical choice model included choice regressors that sorted trials according to choice and/or condition, and several parametric modulators describing the psychological variables across the 6 × 6 task space: environmental threat, number of tokens, *P*(ActBomb), uncertainty and EV (Fig. 1*B*; all psychological variables were modeled separately for the Ap/Av and Ap/Ap conditions). The 6 choice regressors consisted of accept/choose no bomb, reject/choose bomb and explore choices in the Ap/Av versus Ap/Ap condition. Because the main effect of choice and condition × choice contrasts revealed distinct but partially-overlapping clusters in the hippocampus, we followed standard procedures and mutually masked 2 contrasts against each other (at a threshold of *P * = 0.05 uncorrected) in order to identify signals that cleanly showed signals of each kind. Note that the masking procedure is necessary in order to identify signals that are not contaminated by each other. However, this also means that voxels demonstrating a mix of signals do not show up in this analysis. For transparency, we show voxels that demonstrated a conjunction of main effect of choice and condition × choice effects in Supplementary Figure 6*B*. Mutual masking applied only to the hippocampal signals, because this is where the 2 different signals were observed; see Results.

In the chosen and counterfactual value and choice × value difference models, we looked for values signals in the brain that subjects might be using to make strategic choices in our task. The winning behavioral models were used to calculate the values of the chosen and best unchosen options on each trial, as well as the difference between the chosen and counterfactual options (Fig. 5*C*). In the chosen and counterfactual value model, parametric modulators for the chosen and counterfactual value were included (separately for the Ap/Av vs. Ap/Ap conditions) in the first-level GLM. In the choice × value difference model, we modeled the value differences (*V*[Chosen] – *V*[Best Unchosen]) on each trial, splitting trials into whether gambles were rejected on that trial or not (or, in the Ap/Ap condition, whether subjects chose “Bomb” or not).

Further details of model setup are included in the Supplementary materials. Aside from these main fMRI models, further fMRI analysis was conducted to verify that the choice effects noted here were robust to the omission of task-related nuisance variables included in the categorical choice model. Additionally, we constructed region of interest (ROI) estimates of the CA1 and CA3 subfields in order to estimate if the choice-related results corresponded to known anatomical subdivisions within the hippocampus. These analyses are described in full in the Supplementary materials.

Finally, we highlight the fact that all fMRI analysis was conducted in the native space of the group-level template that was constructed using our spatial normalization protocols (see Supplementary materials for full detail). As such, all coordinates reported are not in MNI space. To aim comparison with other studies, all hippocampal clusters are labeled, in coronal slices, with their mm distance relative to the first coronal slice in which the uncus first appears (moving from the posterior to the anterior of the brain). This coronal slice was chosen because the uncus is a major and easily identifiable landmark in the hippocampus, and thus is commonly used in hippocampal segmentation protocols. Note additionally that the activation cluster extends into the space between the hippocampal gray matter and the amygdala in Figure 3*A*; this is likely result of the smoothing procedures that are applied as a standard preprocessing step with fMRI data, combined with the presence of large draining veins in this region of the brain (Lai et al. 1993).

All results were significant at a threshold of at *P *< 0.05 FWE (Family-wise error corrected; with an initial threshold of *P *< 0.001 uncorrected and small-volume correction for the bilateral hippocampus). Where interactions were noted in the voxel-based analysis, we opted to clarify these interactions by examining the simple-effect contrasts within Statistical Parametric Mapping (SPM), as this allowed us to maintain a consistent threshold of *P *< 0.05 FWE. For the choice × value difference model, we employed a functional ROI approach whereby the functional ROIs were not identified using the same model in question (Supplementary Table 7 shows the mean extent to which regressors in the 2 value models are correlated, within subject). For this analysis, we conducted statistical tests on the extracted parameter estimates.

## Results

We first analyzed our behavioral and fMRI data using a model-agnostic approach that focused on choice in the different experimental conditions, without assuming the veracity of any inferred psychological quantities (e.g., values). This analysis aimed to examine whether the hippocampus, during the Ap/Av condition, is selectively implicated in avoidance (i.e., rejecting) or information gathering (exploration). This model-agnostic analysis was then followed up with behavioral modeling and a subsequent model-based analysis of the fMRI data that enabled us to examine hidden informational quantities (i.e., value) that might be used to guide strategic choice in our task, as well as their neural instantiation.

### Dissociating Approach, Avoidance, and Exploration in Approach–Avoidance Conflict

Subjects faced many different combinations of colored backgrounds (indicating environmental threat, ranging from 2/6 to 1) and numbers of activated tokens (ranging from 2 to 12), and had to combine these 2 pieces of information to calculate the probability of an activated bomb (*P*(ActBomb)) on each trial (see Materials and Methods for full detail). These gambles were further encountered in 2 experimental conditions (in a within-subjects design) The approach–avoidance (Ap/Av) condition (Fig. 1*A*, bottom left) invoked an approach–avoidance conflict, because increasing the number of tokens implied a greater magnitude of potential reward (+10 p/token) as well as a greater probability of a substantial loss (a planted bomb exploding, leading to a −120 p loss). In the approach–approach (Ap/Ap) condition, subjects faced the same gambles (i.e., indicating the same *P*(ActBomb)), but without the threat of loss (since they were rewarded for correctly guessing whether there was an activated bomb or not, but were not penalized for being wrong). Thus, any conflict experienced in the Ap/Ap condition stemmed instead from uncertainty about whether the “bomb” or “no bomb” choice options would be more likely to lead to reward. Critically, participants made choices in response to the same gambles (i.e., the same combination of background color and number of tokens) in the Ap/Av and Ap/Ap conditions. This feature of the design allows us to isolate approach–avoidance conflict in our comparison of the 2 conditions, while controlling for working memory requirements, potential use of spatial strategies that might otherwise invoke the hippocampus, and other nonspecific effects. In both conditions, subjects were able to “explore” (i.e., gather more information) the gamble before making a final decision.

The true EV of each choice is shown in Figure 1*B* (bottom left: Ap/Av condition, bottom right: Ap/Ap condition), assuming optimal subsequent behavior in the case of exploration. Although choices in the Ap/Av and Ap/Ap conditions are psychologically distinct from each other, both accepting in the Ap/Av condition and choosing “no bomb” in the Ap/Ap condition indicate a subject's underlying belief that there is no activated bomb on that trial. Similarly, both rejecting in the Ap/Av and declaring “bomb” in the Ap/Ap indicate that a subject believes that there is an activated bomb on that trial. Subjects were first extensively trained on both the Ap/Av and Ap/Ap conditions, separately (see Materials and Methods for more detail). In the scanner, they then performed 12 blocks of trials alternating between Ap/Av and Ap/Ap conditions, with the condition clearly indicated onscreen at all times to prevent confusion (Fig. 1*C*).

**Figure 2.**
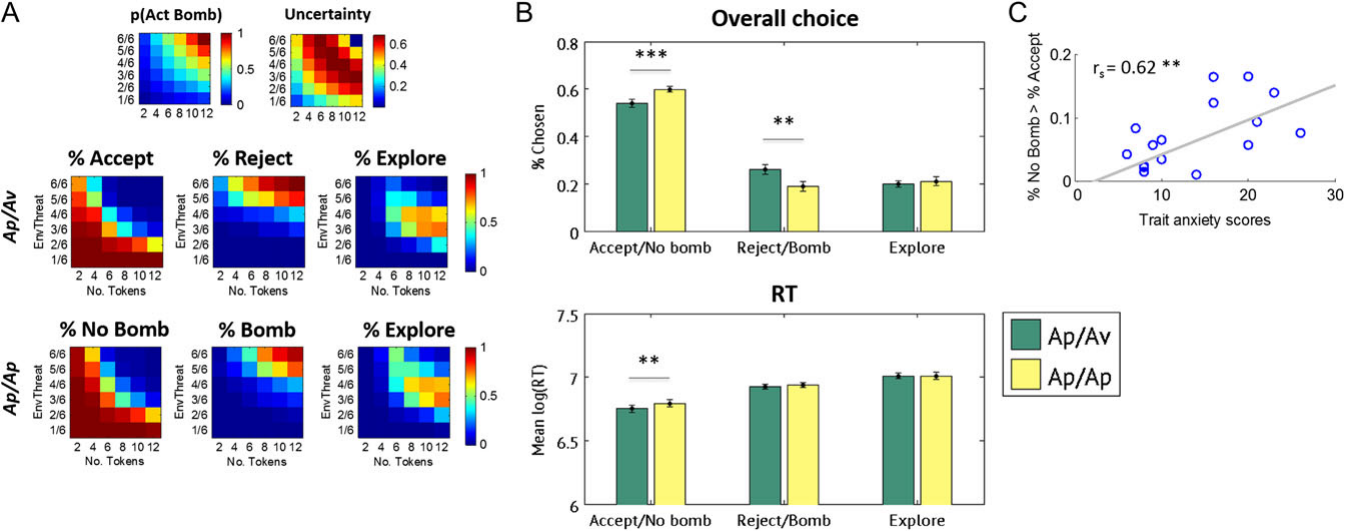
Behavioral choice. Subjects made strategic choices across the 6 × 6 task space (*A*). Accept and reject choices in the Ap/Av condition are analogous to the “no bomb” and “bomb” choices in the Ap/Ap condition, with respect to the subject's underlying belief regarding the presence of an activated bomb. The proportion of the task space in which subjects generally chose “no bomb” in the Ap/Ap was larger compared with the proportion of accepted gambles in the Ap/Av condition (*B*), and subjects were generally faster to accept subjectively safe gambles in the Ap/Av condition, relative to choosing “no bomb” in the Ap/Ap (note that mean RTs are in ms prior to log transformation, with true values ranging from 750 to 1200 ms). Additionally, the extent to which subjects adjusted behavior in response to approach–avoidance conflict was correlated, across all subjects (*n *= 20), with individual differences in anxiety: anxious subjects were more likely to omit accepting gambles on which they thought there was likely to be no activated bomb (*C*).

Figure 2 shows the percentage of accepting, rejecting, and exploring in the Ap/Av and Ap/Ap conditions, as well as the associated response times (RTs). Overall, subjects’ choices appeared to reflect an integration of information from the environmental threat and the number of tokens. The trade-off between accepting versus rejecting (in the Ap/Av condition) and choosing no bomb versus bomb (in the Ap/Ap condition) appeared particularly to track *P*(ActBomb). Analysis with a 2 × 3 (condition × choice) ANOVA revealed a main effect of choice (*F*_2,38_ = 123.23, *P *< 0.001), as well as a condition × choice interaction (*F*_2,38_ = 12.01, *P *< 0.001). Paired *t*-test comparisons confirmed that subjects were more likely to accept (vs. choose “no bomb”), and less likely to reject (vs. choose “bomb”), in the Ap/Ap (compared with the Ap/Av, condition; Fig. 2*B*; Accept/No bomb: *t*(19) = 4.55, *P *< 0.001; Reject/Bomb: *t*(19) = 3.50, *P *= 0.002). This indicates a relative conservatism in accepting gambles in the Ap/Av condition, where incorrect decisions could cause instrumental loss. In contrast, subjects were not more or less likely to explore overall in the Ap/Av versus Ap/Ap condition (*P *> 0.3). As such, the Ap/Av conditions influenced the trade-off between accepting/choosing “no bomb” versus rejecting/choosing “bomb,” leaving the overall levels of exploration relatively similar in the 2 conditions. In order to determine whether the accumulative winnings payoff structure significantly impacted behavior over time, we split trials according to whether they occurred in the initial, middle, and last thirds of the experiment, and compared choice with a condition × choice × third (2 × 3 × 3) ANOVA. No significant effects relating to the section of the experimental session were found (all *P *> 0.4). Subjects were also quicker to accept gambles in the Ap/Av condition, relative to choosing “no bomb” in the Ap/Ap condition (condition × choice: *F*_2,38_ = 2.88, *P *= 0.068; Accept vs. No Bomb: *t*(19) = 3, *P *= 0.005; *P *> 0.3 for other between-conditions choice comparisons. ME choice: *F*_2,38_ = 109.61, *P *< 0.001; ME condition: *P *= 0.069). Additionally, the extent of this trade-off correlated, across all subjects, with individual differences in trait anxiety. The difference between the probability of choosing No Bomb in the Ap/Ap condition and the probability of accepting in the Ap/Av condition was correlated to trait anxiety scores (*r*_s =_ = 0.62, *P *= 0.004). Similar correlations were present with Bomb versus Reject responses, *r*_s _= −0.54, = 0.014, but not for exploration, *P *> 0.1. These results indicate that the behavior elicited by the approach–avoidance conflict in our task is related to the psychological construct of anxiety (as measured by using the STAI questionnaire).

### Frontal, Striatal, and Parietal Regions Support Exploratory Information Gathering

To analyze choice-related signals, we built an fMRI GLM to identify neural regions associated with the different choices in our task. This model-agnostic analysis focused solely on subject's choices, without explicitly modeling the psychological processes that may have been involved in making behavioral choices (e.g., subjective value). In this and all following fMRI models, we focused on the 2000-ms period (indicated by the blue bar in Fig. 1*C*) when subjects evaluated the gambles and made their choices. This first model, referred to as the categorical choice model (see Materials and Methods), categorically modeled choice on each trial, separately for the Ap/Av and Ap/Ap conditions (i.e., 2 × 3, condition × choice). In addition to the 6 regressors of interest, we included nuisance regressors that modeled, on every trial, environmental threat, the number of tokens, *P*(ActBomb), uncertainty, and expected value (EV) of the gamble (Fig. 1*B*, see Materials and Methods for detail). Each variable was modeled separately for the Ap/Av and Ap/Ap conditions. Importantly, these nuisance variables were allowed to compete for variance with the regressors of interest. By including the nuisance regressors, we aimed to remove variance associated with the task-related variables that varied systematically across the task space, in order to identify blood oxygen level-dependent (BOLD) responses uniquely related to choice. As further verification, we examined choice signals using alternative choice models, including a simpler model without these nuisance regressors (Supplementary Fig. 1*A*).

**Figure 3.**
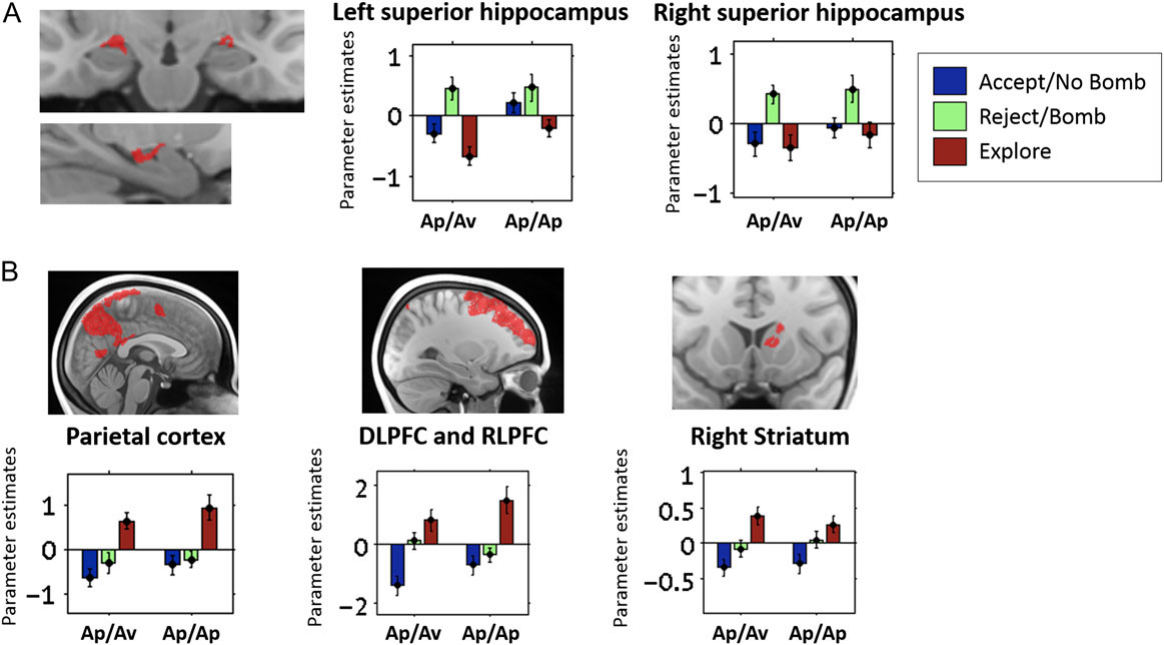
Context-independent choice effects. While the superior anterior hippocampus showed relative “deactivation” when subjects explored (*A*), a network of frontal, striatal, and parietal regions were implicated in exploration (*B*). All clusters are shown at *P *< 0.001, overlaid on the group-level mean anatomical scan. Note that the clusters in the hippocampus shown here and in Figure 4*A* included mutual masking of the main effect of choice and condition × choice contrasts, as is common practice within SPM when examining overlaying effects (see Materials and Methods for more detail). The coronal slice shown in (*A*) is 6.5 mm anterior to the first coronal slice in which the uncus appears (moving from posterior to anterior in the brain; see Materials and Methods for more detail).

We first identified voxels that differentiated between choices without distinguishing between the Ap/Av and Ap/Ap conditions (main effect of choice contrast at the second level). We found significant activation in the bilateral anterior hippocampus, extending into the amygdala (Fig. 3*A*; *P *< 0.05 FWE SVC for the bilateral hippocampus alone, 15.21% of clusters’ voxels in the amygdala; see Supplementary Table 1 for cluster statistics). Follow-up comparisons revealed that the main effect in the hippocampus was driven by high activation when subjects rejected gambles in the Ap/Av condition or similarly chose Bomb in the Ap/Ap condition, and relative deactivation when they chose to explore. No other region in the hippocampus showed greater activation when subjects chose to explore rather than accept/choose no bomb or reject/choose bomb. Instead, exploration was associated with coactivation of frontal, parietal, and striatal regions.

A main effect of choice contrast also revealed activation in a network of regions that included the right striatum, rostrolateral frontopolar cortex (including BA10), middle frontal gyrus (including BA46), superior frontal gyrus (dorsolateral prefrontal cortex; DLPFC), striatum and parietal cortex; Figure 3*B*; see Supplementary Table 1 for full statistics. Follow-up contrasts revealed a consistent pattern of greater activation when subjects chose to explore, compared with when they chose to accept/choose no bomb or reject/choose bomb, in all these regions (see Fig. 3*B* for parameter estimates from these clusters). No regions showed a significantly greater BOLD response when subjects accepted gambles/chose no bomb (compared with rejecting/choosing bomb or exploring), which may have resulted from our controlling for quantities like EV and number of tokens in the fMRI model.

Although we did not find evidence for hippocampal activation when participants chose to explore, the regions identified are in line with existing findings on exploration in decision-making. One alternative possibility that arises from Gray and McNaughton's (2000) hypothesis is that the hippocampus may be specifically recruited in exploration in response to approach–avoidance conflict rather than exploration more generally. To examine this possibility, we directly contrasted exploration in the Ap/Av versus Ap/Ap condition (ignoring accept/choose no bomb and reject/choose bomb choices), again using the voxel-based approach. This specific contrast did not reveal any significant activation in the hippocampus, or elsewhere in the brain. Thus, these results do not support the hypothesis that the hippocampus is involved in initiating exploratory risk-assessment behavior in the context of approach/avoidance conflict, as hypothesized by Gray and McNaughton (2000). Instead, exploration in both the Ap/Av and Ap/Ap conditions was supported by a network of regions commonly implicated in decision-making and executive control (Daw et al. 2006; Badre et al. 2012). These results indicate that exploration in an approach–avoidance context is not qualitatively different from exploration in a broader decision-making context.

### Subregions of the Anterior Hippocampus Are Specifically and Selectively Recruited During Avoidance in An Aversive Context

**Figure 4.**
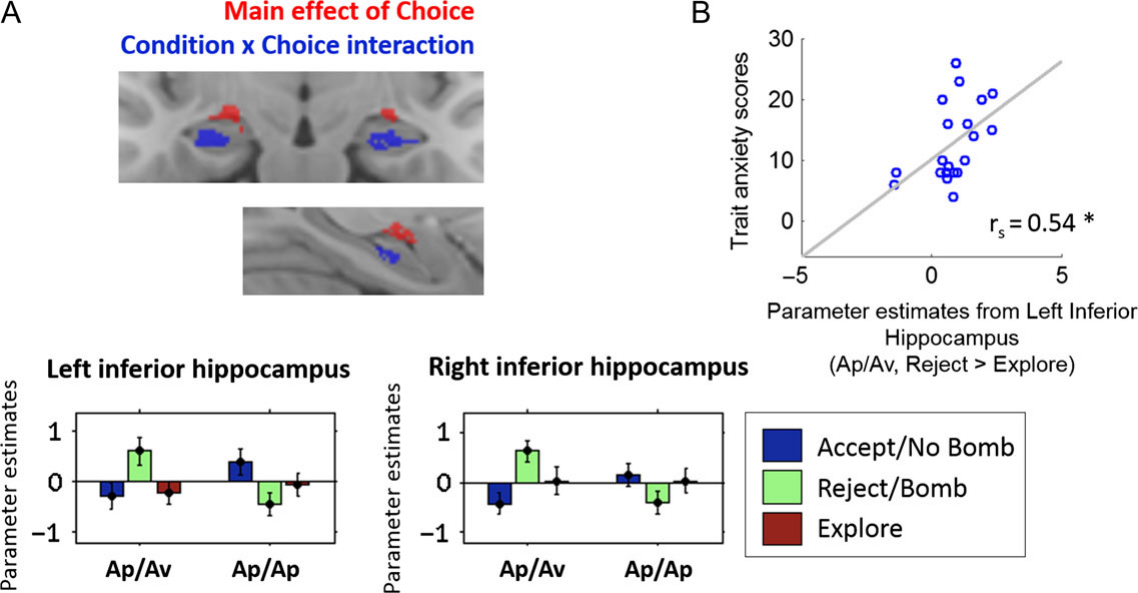
The anterior hippocampus supports behavioral avoidance. Voxels in the inferior anterior hippocampus discriminated between rejecting gambles in the Ap/Av condition and choosing “bomb” in the Ap/Ap condition (*A*). While both these trial types were associated with subjects believing that there was likely to be an activated bomb, the Ap/Av condition alone invoked an additional avoidance response, due to the potential for instrumental loss. In contrast, the superior anterior hippocampus (*A*, red; extending slightly into the amygdala) failed to discriminate between these 2 trial types, despite their markedly different psychological impact. Additionally, the strength of this response in the left inferior anterior hippocampus correlated across all subjects (*n* = 20) with measures of anxiety (*B*). The coronal slice shown in (*A*) is 5.2 mm anterior to the first coronal slice in which the uncus appears.

Our previous analysis did not identify any significant voxels that distinguished exploration in the 2 conditions. Using the same fMRI model, we next examined the condition × choice interaction contrast to identify brain regions that responded differentially to choice in the Ap/Av and Ap/Ap conditions. This is the key contrast of interest for our study, as we were interested in examining hippocampal contributions to choice that differed in the context of approach–avoidance conflict. We found significant clusters in the bilateral inferior anterior hippocampus (Fig. 4*A*, blue; *P *< 0.05 FWE SVC for the bilateral hippocampus; see Supplementary Table 1 for cluster details and Supplementary Figure 6*A* for comparison of this ROI with the anatomically defined amygdala).

To identify which choices were involved in driving this interaction, we looked for activation in these clusters for the contrasts relating to condition × reject/choose bomb versus explore, condition × accept/choose no bomb versus reject/choose bomb, and condition × accept/choose no bomb versus explore. The effects were driven by differences in rejecting/choosing bomb versus exploring in the 2 conditions (at *P *< 0.001, significant activation in these clusters observed for the condition × reject/choose bomb vs. explore contrast, but not the others). Specifically, while these regions showed greater activation when subjects rejected gambles in the Ap/Av condition, they were relatively deactivated when subjects faced the analogous decision in the Ap/Ap condition (Fig. 4*A*). This indicates a specific role for inferior anterior hippocampus when subjects act to avoid a possible loss, as distinguished from acknowledging the presence of a hypothetical threat that could not harm them. Additionally, the strength of this left hippocampal response (reject > explore in the Ap/Av condition) was positively correlated across subjects with individual differences in anxiety (Fig. 4*B*; trait anxiety: *r*_s_ = 0.54, *P *= 0.014; *P * > 0.1 for the right hippocampal cluster).

To avoid confounds between choice effects and task-related quantities, our fMRI model included regressors for various task-related psychological variables (including EV and *P*(ActBomb); Fig. 1*B*). This allowed us to identify the hippocampus’ role in the behavioral avoidance of aversive threat beyond the passive tracking of *P*(ActBomb). In a confirmatory analysis, using a simpler categorical choice model without the nuisance variables, we found the same choice-related results (Supplementary Fig. 1*A*). This demonstrates that the choice-related results identified in the main analysis are not an artifact of the nuisance regressors that we controlled for in the full choice model. To further verify these results, we also conducted further analysis examining choice in a more restricted portion of the task space. We were able to verify that the anterior hippocampus robustly distinguished between avoiding in the Ap/Av and Ap/Ap conditions, even when analysis was restricted to the portion of the task space in which *P*(ActBomb) remained relatively unchanged (Supplementary Fig. 2*A*).

Finally, we considered the possibility that the results in the inferior anterior hippocampus may have been confounded by greater subjective (rather than objective) uncertainty when subjects rejected in the Ap/Av condition, relative to choosing bomb in the Ap/Ap condition. We examined choice entropy as an index of subjective uncertainty across the different choices (Supplementary Fig. 2*B*) and showed that our findings are unlikely to be confounded by greater subjective uncertainty in the Ap/Av condition. Additionally, the results in the inferior hippocampus were robust to the inclusion of RTs (a proxy for trial-to-trial subjective uncertainty) in the fMRI model (i.e., as regressors of no interest; see Supplementary Fig. 2*C* for detail). These further analyses rule out the possibility that the inferior hippocampal results were merely reflective of differences in the subjective uncertainty between these 2 choice types.

We noted that the clusters showing a main effect of choice and those showing a condition × choice interaction were consistently segregated within the anterior hippocampus: voxels showing main effect of choice were relatively superior to voxels showing a condition × choice interaction, on both the left and the right (Fig. 4*A*), as well as in individual subjects’ native space (Supplementary Fig. 1*B*). Comparing our functional clusters with anatomical atlases and reference images in which hippocampal subfields had been manually segmented according to anatomical features (Wisse et al. 2012; Duvernoy 2013), we noted that the voxels that demonstrated a main effect of choice (Fig. 4*A*, red) overlapped bilaterally with the CA3 subfield of the hippocampus, with extensions into the amygdala. On the other hand, voxels showing a condition × choice interaction overlapped mostly with the CA1 subfield on the right and CA1/DG on the left, with extensions into the subiculum, bilaterally. A similar overlap with the CA1 subfield was observed in our additional analysis focusing on those trials in which rejecting choices traded off with exploration (Supplementary Fig. 2*A*). As such, robust discrimination between rejecting in an instrumentally aversive versus neutral context emerged most robustly in the inferior hippocampus, where the CA1 cells are located. However, despite their close proximity, voxels in the superior hippocampus and possibly corresponding to CA3 demonstrated a distinct pattern of choice-related effects: activation when subjects believed there was an activated bomb without distinguishing between the Ap/Av and Ap/Ap conditions (Fig. 3*A*).

Although the spatial resolution of our functional data limited our ability to confidently segment all the hippocampal subfields, we were able to build estimated anatomical ROIs of CA3 and CA1 based on the group anatomical image (Supplementary Fig. 3*A*; see Supplementary materials for detail). Using these ROIs, we then observed that a similar main effect of choice and condition by choice interaction could be found in the left CA3, and bilateral CA1, respectively (Supplementary Fig. 3*B*; see Supplementary materials for more detail). These findings, while exploratory, highlight the possibility that the different functional signals within the anterior hippocampus may map onto anatomically distinct subregions, and suggest that threat avoidance specifically may implicate the inferior sections of the anterior hippocampus, where CA1 neurons are typically located.

### Behavioral Modeling

The results implicate a network of frontal, parietal, and striatal regions in exploratory risk assessment. Additionally, they implicate the anterior hippocampus in the avoidance of aversive outcomes. In addition to these model-agnostic choice effects, we were interested in identifying value-related signals that underpinned subjects’ decisions to accept/choose no bomb, reject/choose bomb or explore when faced with the different gambles. To calculate these value signals, we fit a family of decision models to subjects’ choices, parameterizing deviations from behavior that would be optimal according to the objective contingencies. Models calculated values on each trial for each of the 3 possible actions (accept/choose no bomb, reject/choose bomb, explore), which were then translated into choice probabilities via a softmax rule. Potential sources of suboptimality (e.g., loss aversion, mis-estimation of environmental threat) were also considered in our model space (see Materials and Methods for more detail, and Supplementary materials for full detail of all possible parameters considered). We used Bayesian model comparison to select the model that best captured observed behavior.

**Figure 5.**
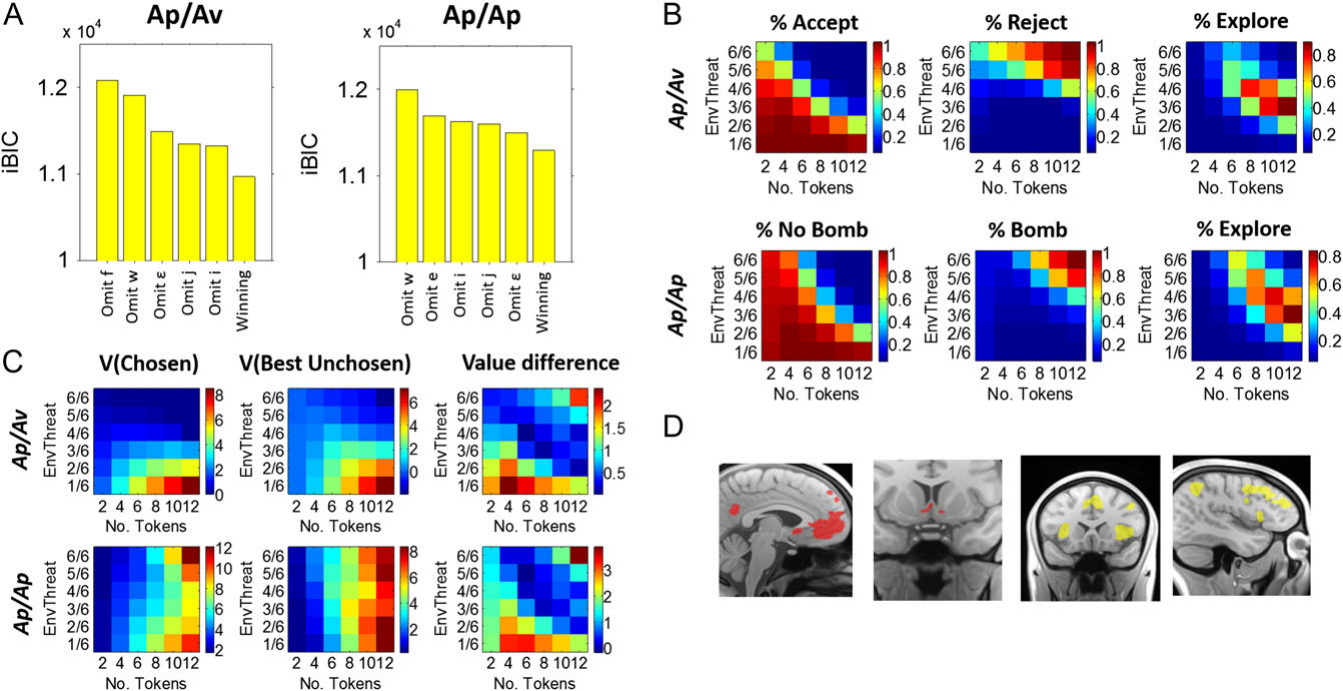
Behavioral modeling and value signals. Bayesian model comparison was used to select the winning model from a large model space; BICs for selected models are shown in (*A*). The winning models were able to closely reproduce the overall pattern of choice observed in both the Ap/Av and Ap/Ap conditions (*B*; see Fig. 2*A* for observed behavior). Values relating to the chosen and counterfactual options, as well as the value difference between the 2, were employed in our fMRI analysis (*C*). The vmPFC, ventral striatum, and other regions tracked this value difference positively (*D*, red), whereas other regions like the parietal cortex, inferior frontal gyrus, and DLPFC tracked *V*(Best Unchosen) > *V*(Chosen) (*D*, yellow).

In both the Ap/Av and Ap/Ap conditions, the winning model (detailed below) explained the data very well (pseudo-*r*^2^= 0.64 and 0.62 for Ap/Av and Ap/Ap, respectively). BICs for the winning model compared with adjacent models in the model space (i.e., that omit individual parameters) are presented in Figure 5*A* (see Supplementary Fig. 4*B* for BIC values across the entire model space considered). Additionally, simulated choice predicted by the winning models (Fig. 5*B*) closely reproduced the pattern of observed behavior (see Fig. 2*A* for comparison). Reflecting the ability of the model to predict choice, simulated behavior reflected the same relationship between individual differences in choice and trait anxiety as observed in the data (predicted difference between the probability of choosing no bomb and the probability of accepting was correlated with trait anxiety; *r*_s_ = 0.51, *P *= 0.02).

The winning models showed that subjects slightly undervalued or underestimated the magnitude of the fixed loss in our task (described by the *f* parameter; mean subjective value = −10.03 tokens; true = −12 tokens; see Materials and Methods and Supplementary materials for detail on all parameters). At the time of their initial choice, subjects over-valued accepting gambles/choosing no bomb after exploration revealed no bomb, in both the Ap/Av and Ap/Ap conditions (described by the *i* parameter). Subjects’ underlying estimate of the environmental threat associated with each background color were distorted relative to the true probabilities (described by the *j* parameter), but environmental threat was not systematically under- or over-estimated by the group as a whole (see Supplementary Fig. 4*C* for the average task space experienced by subjects). Subjects were more likely to explore than was optimal, and an exploration bonus linked to uncertainty was found in both the Ap/Av and Ap/Ap conditions (described by the *w* parameter). This means that subjects were more likely to explore on trials in which uncertainty was high (see Supplementary Materials and Methods for more detail on other candidate variables that were considered as a potential modulator of exploration).

### Hippocampal Values Signals Are Magnified in the Context of Approach/Avoidance

Signals relating to the value of chosen and counterfactual (i.e., unchosen, competing) alternatives are thought to be important in guiding choice. Accordingly, we built an fMRI model (referred to as the Chosen and Counterfactual Value model in the Materials and Methods), with the values of the chosen and best unchosen options (as predicted using the winning behavioral models; Fig. 5*C*) as parametric modulators on each trial. Consistent with previous work, we found neural signals that tracked subjective chosen values (i.e., *V*[Chosen] > *V*[Best Unchosen]) in ventromedial prefrontal cortex (vmPFC) and ventral striatum (Fig. 5*D*; see Supplementary Table 4 for full list of activation and statistics). Additionally, regions like the dorsomedial prefrontal cortex and DLPFC extending to the frontal pole were observed to track counterfactual values (*V*[Best Unchosen] > *V*[Chosen]).

**Figure 6.**
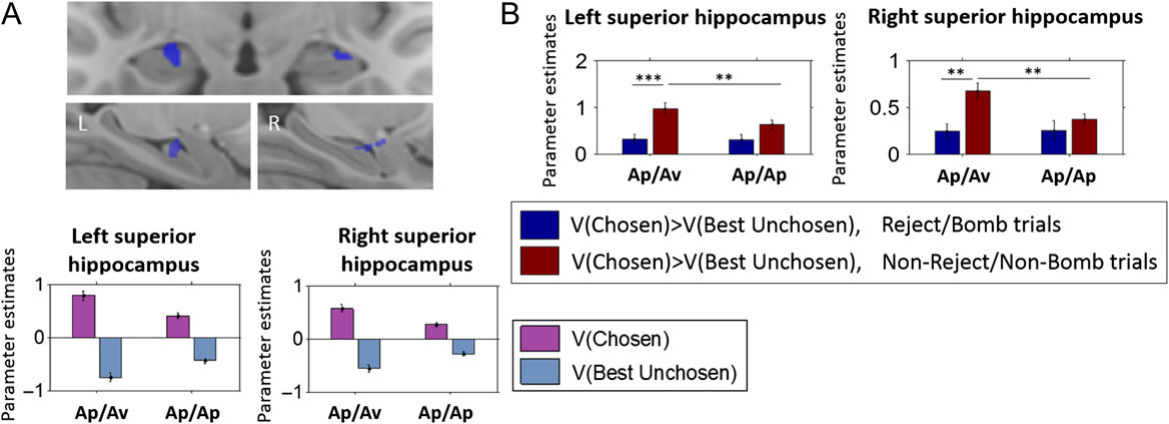
Hippocampal value signals. Value signals in the superior hippocampus (extending slightly into the amygdala) were potentiated in the Ap/Av condition, compared with the Ap/Ap (*A*). These potentiated value signals were driven by trials on which subjects omitted to reject gambles in the Ap/Ap condition (*B*). In the Ap/Av condition, stronger tracking of value (i.e., comparing the Ap/Av and Ap/Ap conditions) was observed on trials in which subjects accepted or explored gambles in the Ap/Av condition, compared with 1) choosing “no bomb” or exploring in the Ap/Ap condition, as well as compared with 2) rejecting in the experimental condition (note the interaction is a statistical trend for the left hippocampus). The coronal slice shown in (*A*) is 6.5 mm anterior to the first coronal slice in which the uncus appears, and the sattigal slices, labeled L (left) and R (right), are respectively 28.6 mm left and 32.5 mm right of the midline.

To identify neural value signals that were modulated by the approach–avoidance context, we included these same value regressors in a second-level 2 × 2, condition (Ap/Av vs. Ap/Ap task) × value (*V*[Chosen] vs. *V*[Best Unchosen]) model, and examined the interaction contrast. Only the superior hippocampus, extending into the amygdala (35.21% of clusters’ voxels in the amygdala), showed a significant interaction between value and task (Fig. 6*A*; SVC for the bilateral hippocampus; left: peak at −21.8, 6.7, 12.9, 183 voxels, *T* = 4.05, peak FWE *P *= 0.037; right: peak at 19.2, 11.0, 11.8, 183 voxels, *T* = 4.32, peak FWE *P *= 0.018). Examination of the follow-up contrasts within this interaction indicated that this interaction was driven by stronger tracking of value differences (i.e., *V*[Chosen] > *V*[Best Unchosen]) in the Ap/Av compared with the Ap/Ap condition.

Since our results implicated hippocampus in rejecting gambles, we conducted a final analysis to examine whether these potentiated hippocampal value signals were modulated by choice. We built a final fMRI model that included value differences (i.e., *V*[Chosen] > *V*[Best Unchosen]; Fig. 5*C*) separately depending on whether the gamble was rejected or not on each trial (referred to as choice × value difference in the Materials and Methods). Note that we focused on value differences rather than *V*(Chosen) and *V*(Best Unchosen) separately, since *V*(Chosen) was always equivalent to 0 when subjects opted to reject in the Ap/Av condition. Within this first-level model, we focused on the functional ROI that had been identified in the previous analysis (i.e., Fig. 6*A*), extracting parameter estimates from these superior hippocampal clusters, and analyzing them with a 2 × 2 (condition × choice) ANOVA. Note that the choice by value difference by condition contrast examined here is orthogonal to value difference by condition contrast used to define the functional ROIs.

This final analysis revealed an interaction between condition and choice and value difference (see Table 1 for full statistics; note the interaction remains a statistical trend on the left, although ROI × choice × value type interaction, *P * > 0.9). The interaction was driven by value difference signals that were significantly stronger on nonrejected trials in the Ap/Av condition (Fig. 6*B*), compared with both the signals on nonreject trials in the Ap/Ap condition, as well as to the signals on reject trials in the Ap/Av condition. These results suggest that superior hippocampal value signals were potentiated specifically on trials in which subjects omitted to reject in the Ap/Av condition but not in the Ap/Ap condition. Motivated by the extension of the value signals into the amygdala (Fig. 6*A*), we additionally conducted an exploratory analysis in order to examine the role of the amygdala in signaling value along with the hippocampus, and found qualitatively similar value signals in the right amygdala (albeit with a nonsignificant interaction) but not the left (see Supplementary Fig. 6*E* for full detail).

**Table 1:** Hippocampal value signals by condition and choice

	Left	Right
2 × 2 (condition × choice) ANOVA (df = 1,19)		
ME condition	*F* = 3.70, *P *= 0.070	*F* = 4.16, *P *= 0.056
ME choice	*F* = 12.55, *P *= 0.002**	*F* = 7.39, *P *= 0.014*
Condition × choice	*F* = 3.25, *P *= 0.088	*F* = 5.10, *P *= 0.036*
Simple effects (df = 19)		
Ap/Av, reject versus others	*t* = 4.26, *P *< 0.001***	*t* = 3.69, *P *= 0.002**
Ap/Ap, bomb versus others	*t* = 1.90, *P *= 0.072	*P *> 0.01
Reject versus bomb (Ap/Av vs. Ap/Ap)	*P *> 0.01	*P *> 0.01
Accept/explore versus no bomb/explore (Ap/Av vs. Ap/Ap)	*t* = 3.33, *P *= 0.004**	*t* = 3.8, *P *= 0.001**

The potentiation of value signals on nonreject trials in the Ap/Av condition relative to the Ap/Ap condition is consistent with a role for the hippocampus, potentially aided by the right amygdala, in monitoring nonavoidant choices in the context of approach–avoidance conflict. Interestingly, these value signals were observed in the superior hippocampus, which had not distinguished between rejecting in the Ap/Av and Ap/Ap conditions using the categorical choice model. The findings indicate that value signals are potentiated in the superior anterior hippocampus when subjects face conditions in which threats are instrumentally relevant, but perhaps to a degree that is insufficient to motivate avoidance. In contrast, when actions are taken to avoid instrumental threats, the superior hippocampus shows no such potentiation in its value signals, and the inferior hippocampus instead is implicated in a choice-related manner.

## Discussion

We examined hippocampal contributions to approach–avoidance conflict by developing a novel decision-making task that separated behavioral avoidance (reject) from the decision to gather more information (explore). We compared behavior and neural signals in the context of Ap/Av conflict to behavioral and neural signals in the context of Ap/Ap conflict. Whereas the former involves conflicting approach and avoidance tendencies because of the possibility of a big loss, any conflict or ambiguity about the best course of action in the latter stems from conflicting approach impulses. We observed 2 different functional signals in the anterior hippocampus related to behavioral avoidance. First, the inferior anterior hippocampus was selectively activated when subjects acted to avoid a potential loss in the context of approach–avoidance conflict, to a degree that covaried with individual differences in trait anxiety in our sample of 20 subjects. Second, the superior anterior hippocampus demonstrated value signals that were potentiated by an approach–avoidance context, specifically on trials in which subjects omitted to avoid potential threats.

These findings support the idea that the hippocampus is involved in both monitoring and behavioral control in the context of approach–avoidance conflict. Such conflict between approach and avoidance impulses is contrasted with other forms of conflict that require animals to decide between conflicting courses of action in the absence of Pavlovian conflict (e.g., in the Ap/Ap condition). In the superior hippocampus, value signals were potentiated in an approach–avoidance context, specifically for trials in which subjects omitted to reject (in the Ap/Av condition). This signal may reflect hippocampal monitoring of potential outcomes, under conditions where threats are instrumentally relevant but insufficiently great to motivate actual avoidance. In contrast, the choice-related effects in the inferior hippocampus (i.e., greater activation when subjects reject in an approach–avoidance context) are consistent with a role for this region in coordinating avoidant action when threats are sufficiently great for avoidance to be an imperative.

Dual monitoring and control modes have been previously suggested in the literature (Gray and McNaughton 2000), but are difficult to demonstrate in rodents, because experimental indices of threat awareness (e.g., freezing) are tightly linked to behavioral control. Here, we identify expected outcomes as a potential quantity that may be tracked specially by the hippocampus in order to implement monitoring in an approach–avoidance context. By employing computational methods that quantify the subjective values underlying choices, we were able to examine hippocampal contributions to approach–avoidance processing outside the limited set of circumstances that merit a behavioral response.

A network of regions including the vmPFC and ventral striatum demonstrated characteristic value difference signals (i.e., tracking *V*[Chosen] > *V*[Best Unchosen]; Fig. 5*D*), which may reflect the brain's sensitivity to quantities that are ultimately used to guide strategic choice in our task. However, voxels in the superior anterior hippocampus uniquely showed an interaction between value and Ap/Av condition. This indicates that comparable changes in EVs in the Ap/Av condition resulted in a steeper change in activation in the superior hippocampus, compared with elsewhere in the brain (Fig. 6*A*). Previous work indicates that values of competing options are represented in the brain, and that competitive interactions between such neural representations are important in determining behavioral choice (Hayden et al. 2009). In this context, the hippocampus may serve to potentiate value signals that invoke approach–avoidance conflict, thereby sharply defining the brain's representation of competing alternatives in situations where unfortunate decisions may entail instrumental loss. Hippocampal representations of fictive, counterfactual experiences have been noted to play an important role in decision-making (e.g., Wimmer and Shohamy 2012; Redish 2016). One intriguing possibility is that hippocampal tracking of approach–avoidance outcomes may relate to its broader role in representing episodic experiences in rich contextual detail (Tulving 1985; Burgess et al. 2002). For example, situations of conflict between approach and avoidance may implicitly motivate subjects to represent (fictive) potential outcomes more strongly and vividly. Alternatively, pattern completion processes within the hippocampal CA3 may result in a wider network of episodic representations being reactivated in the brain (Horner et al. 2015), leading to stronger or more vivid representations of potential outcomes.

The categorical choice signals observed in the hippocampus indicate that the hippocampus is recruited for avoidance in an Ap/Av context, rather than in relation to exploration. The strength of the avoidance-related response also correlated, across all subjects, with individual differences in trait anxiety (albeit in a relatively small sample size of *n* = 20; Fig. 4*B*). These findings replicate previous work that had implicated the human hippocampus in the processing of mixed-valence stimuli (O'Neil et al. 2015). Although the hippocampal cluster that responded to mixed-valence stimuli in O'Neil et al.’s study did not show any specific involvement in avoidance or approach, we note that our experiment has roughly 10× more trials than that of O'Neil and colleagues, which gave us more statistical power to detect a specific involvement of the hippocampus in avoidance. Additionally, O'Neil et al. also report another cluster in the hippocampus that was recruited when subjects approached both conflict and nonconflict stimuli. Given the recruitment of this cluster in both conflict and nonconflict conditions (i.e., in response to mixed-valence as well as single-valence stimuli), it is likely that this activation reflects hippocampal involvement in other incidentally invoked cognitive processes unrelated to the Ap/Av conflict (e.g., reward anticipation; Murty et al. 2016). In support of a hippocampal role for avoidance, we note that lesions to the ventral hippocampus in rats produce impulsivity and deficits in inhibitory control, even in the absence of an aversive context (Bannerman et al. 2003; Pentkowski et al. 2006; Abela et al. 2013). Similarly, human patients with anterior hippocampal lesions show decreased passive avoidance and behavioral inhibition when performing a human analog of the tests typically used to study anxiety in rodents (Bach et al. 2014). As such, our data are consistent with the suggestion that the anterior (as opposed to the posterior) hippocampus is particularly involved in the processing of affective states such as anxiety (Fanselow and Dong 2010).

The function of the hippocampus in exerting inhibitory control on motor plans may indeed explain why approach–avoidance conflict, which invokes incompatible motor plans, is such a key feature in the rodent anxiety tests that have so convincingly implicated this structure (Gray and McNaughton 2000). An open question for future work concerns the extent to which the hippocampus is implicated in behavioral inhibition that does not conflict with any ongoing approach impulses. Straightforward avoidant responses (e.g., withdrawing from pain) and paradigms that involve explicit expectations of imminent aversive experiences may instead tap into other aspects of fear responding that recruit other structures in the brain. Additionally, future work should aim to identify the fundamental computations performed in the hippocampus to support avoidance in the context of Ap/Av conflict and test the relationship of those to other cognitive functions supported by the hippocampus.

Although the spatial resolution of our MRI data precludes formal quantification of the signals in different hippocampal subfields, the segregation of choice-related signals mirrored the spatial segregation of the different subfields in the anterior hippocampus. In the analysis of choice-related signals, the inferior anterior clusters that robustly distinguished between rejecting in the Ap/Av and choosing “bomb” in the Ap/Ap conditions (Fig. 4*A*, blue) overlapped with the CA1 subfield. In contrast, the superior hippocampus, overlapping with the CA3 subfield, failed to distinguish between these analogous choices in the different conditions (Fig. 4*A*, red), despite the close proximity of these voxels to the voxels in the inferior hippocampal clusters. We caution that our mapping of inferior and superior hippocampal signals onto the underlying hippocampal subfields should be considered exploratory, given the limited spatial resolution of our fMRI data. Nonetheless, we include these results in order to highlight hippocampal subfield interactions as a potentially productive focus for future work. Existing work on human hippocampal contributions to anxiety has tended to focus on orthogonal differences between anterior and posterior segments while our results point toward the existence of different functional signals within the anterior hippocampus itself, and suggest that threat avoidance specifically may implicate the inferior sections of the anterior hippocampus, where CA1 neurons are typically located.

One possibility is that processing within the hippocampal circuit may transform a neural signal that relates to monitoring into a signal that relates to categorical choice. Although value signals are ubiquitous in the brain, in the context of approach–avoidance conflict, the CA3 subregion of the hippocampus may access and potentiate this value signal, possibly via pattern completion mechanisms (which are thought to be implemented via the recurrent circuitry of this part of the brain; see Knierim and Neunuebel 2016 for recent review). These potentiated value signals may allow for a categorical decision variable to emerge in downstream CA1, a region of the brain that has previously been implicated in the processing and detection of novelty and associative mismatch (Hasselmo et al. 1996; Kumaran and Maguire 2006, 2007). Such signal transformation between CA3 and CA1 subfields could be mediated by the strength of activation in CA3 neurons, or could alternatively rely on modulation by inputs from other neural regions, such as the amygdala or prefrontal cortex. In fact, exploratory analysis revealed similarly potentiated value signals in the right amygdala (Supplementary Fig. 6*E*), and others have also noted similar signals in anterior cingulate (Amemori and Graybiel 2012). In rodents, both amygdala and prefrontal regions project bidirectionally to the ventral hippocampus, the rodent homolog of the human anterior hippocampus, and amygdala inputs to the ventral hippocampus bidirectionally modulate anxiety-related behavior (Felix-Ortiz et al. 2013). Such a circuit-level mechanism for converting value signals in upstream regions into categorical choice signals in downstream subregions could plausibly underpin a hippocampal role in both monitoring and active behavioral control.

Information seeking (exploration) did not elicit positive hippocampal activation in our task in either the Ap/Av or Ap/Ap conditions. Indeed, we did not find any region in the brain that distinguished between exploring in the Ap/Av versus Ap/Ap conditions. The lack of an exploration-related condition × choice interaction in the hippocampus suggests that approach–avoidance conflict per se does not influence the extent to which information seeking positively engages the hippocampus. Further, our results indicate that information seeking in an approach–avoidance context is qualitatively similar to exploration that occurs in a broader context. In both conditions, we found that uncertainty-based exploration engaged a network of regions that included the DLPFC, frontal pole and striatum, regions widely associated with this function (Daw et al. 2006; Badre et al. 2012) in a variety of probabilistic decision-making studies. By contrast, the involvement of the hippocampus in exploration has more frequently been seen in tasks that emphasize the use or maintenance of acquired information (e.g., Wang and Voss 2014).

Different types of exploration might engage hippocampal-dependent processes to varying extents, and so elicit more or less activity in this region. For example, cognitive processes like memory, constructive imagination, or forward simulation are known to involve the hippocampus in situations that do not involve information seeking (Kumaran and Maguire 2007; Hassabis and Maguire 2009; Redish 2016). Thus, types of exploration that involve these processes might lead to hippocampal activation (Simon and Daw 2011; [Bibr bhw378C9][Bibr bhw378C10]; Wang and Voss 2014), whereas ones that arguably do not, might not (Daw et al. 2006; Badre et al. 2012). One speculative possibility is that, in our experiment, hippocampal deactivation when subjects explored (Fig. 3*A*) may have reflected subjects disengaging from representing the probabilistic outcomes associated with the gambles in an episodic manner, with exploration framed as a decision to defer difficult choice in lieu of active information gathering activities.

By combining careful experimental design, behavioral modeling and fMRI, we have been able to parse hippocampal contributions to avoidance and exploratory risk assessment when human subjects faced an approach/avoidance conflict, while controlling for spatial and mnemonic processes that may also be invoked. Our results demonstrate that the hippocampus supports behavioral avoidance as opposed to exploration in the context of approach–avoidance conflict. Additionally, we identify different functional signals within the anterior hippocampus that relate to threat monitoring and avoidance. While the spatial resolution of our data precludes confident conclusions about subfield activations in our task, the data nevertheless points toward the CA1 region in particular as a potentially productive focus for future work.

## Supplementary Material

Supplementary Data
