# Supplementary material for: Parsing the Role of the Hippocampus in Approach–Avoidance Conflict
Source: Cereb Cortex. 2016 Dec 18;27(1):201–15. doi: 10.1093/cercor/bhw378 (PMC5939226; doi:10.1093/cercor/bhw378)
Supplement: Supplementary Data [file supplementary_v5-13.docx]

**Supplemental Figures**

**Figure S1: Choice related results**


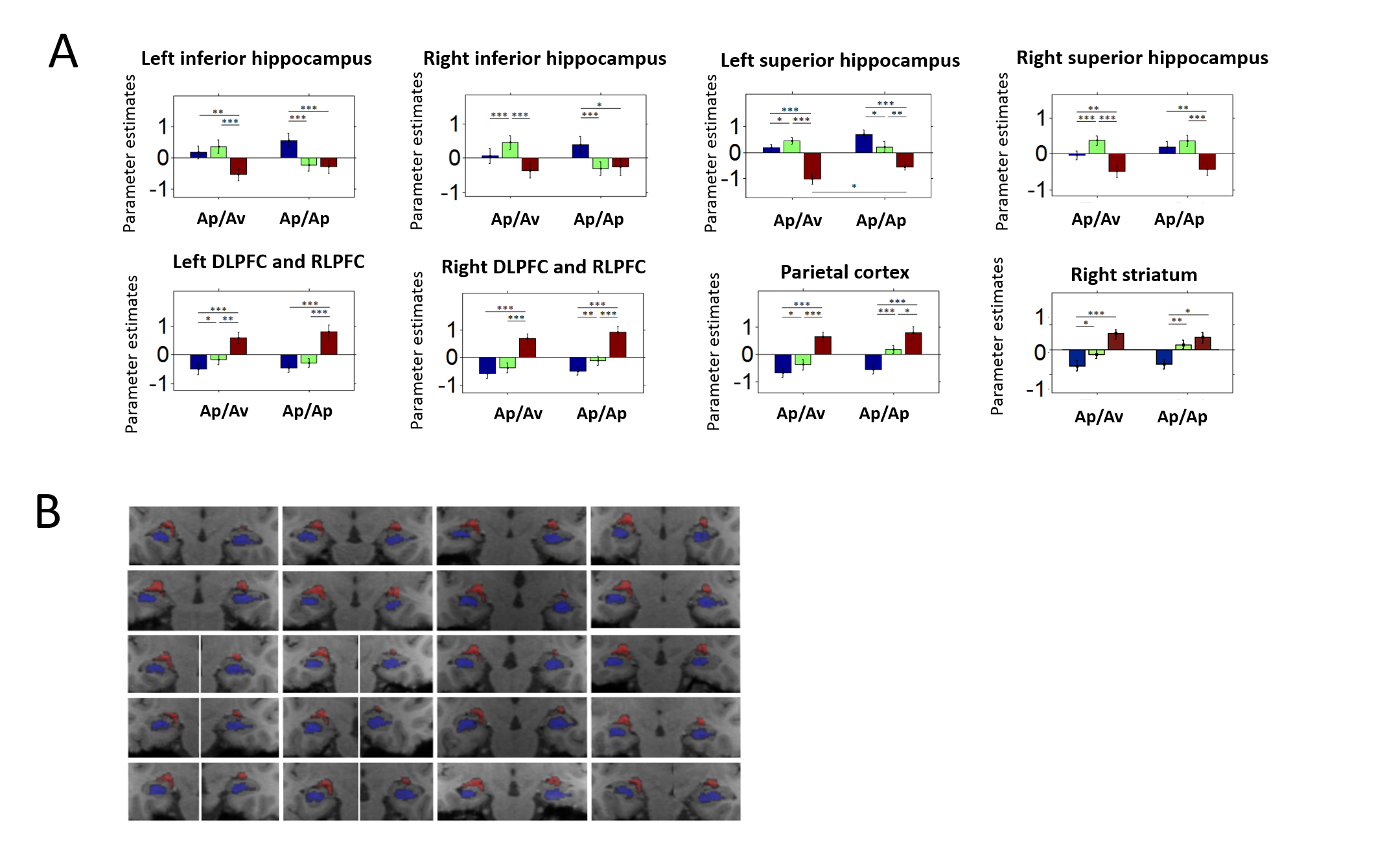

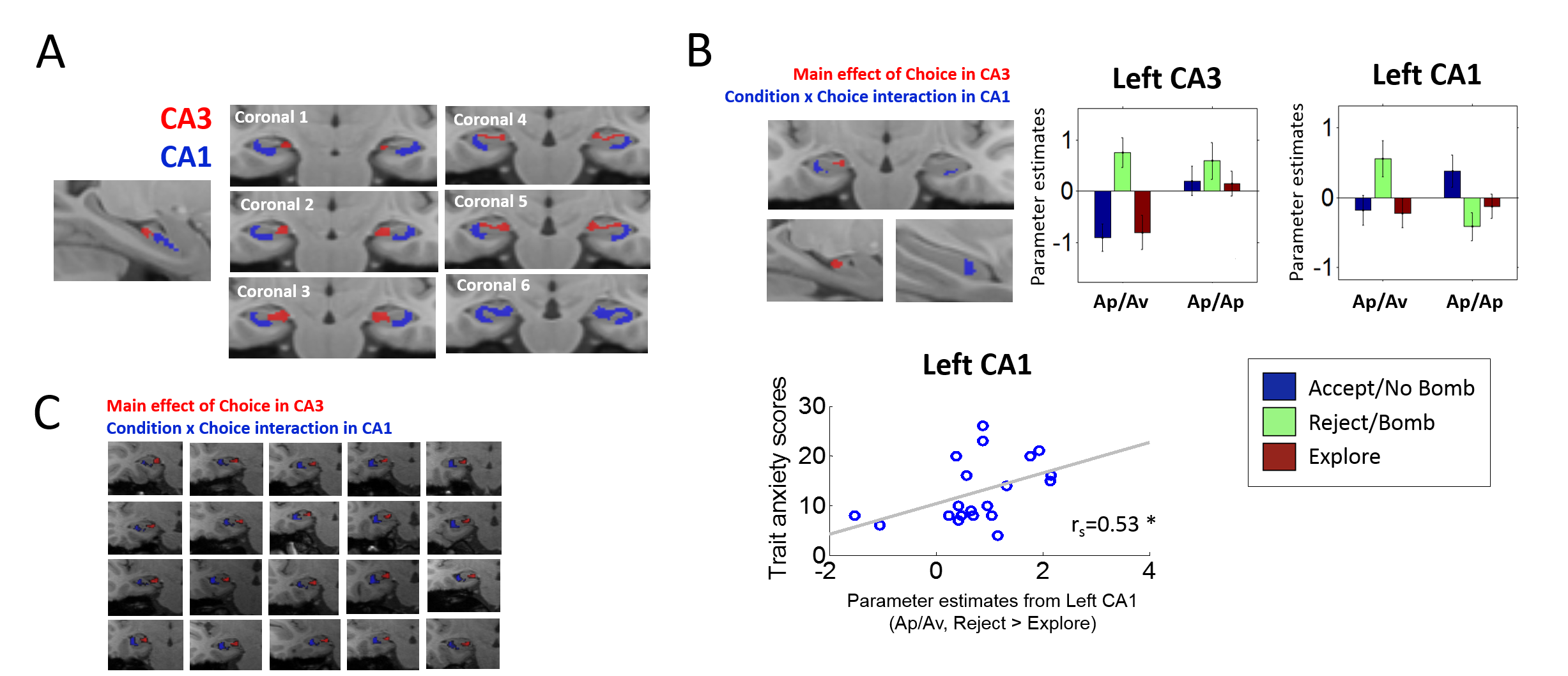


(A) Patterns of activation in the functional ROIs identified using the categorical choice fMRI model (i.e., Figure 3-4), as tested in an emptier choice fMRI model that omitted regressors for the various manipulated psychological variables (‘*choice only’* model, see Supplemental Materials and Methods for detail). Analyzing extracted parameter estimates from fMRI model recapitulated the pattern of results reported in the main text (see Table S2 for statistics): the inferior hippocampal ROIs showed a condition x choice interaction, driven by a significant difference between rejecting/choosing bomb and exploring in the Ap/Av condition, but not in the Ap/Ap. Both the superior hippocampal ROIs showed a main effect of choice, with significant differences between the parameter estimates for rejecting/choosing bomb and exploring, in *both* the Ap/Av and Ap/Ap conditions. The left ROI additionally showed a condition x choice interaction, which appeared to be driven by a significant difference in the parameter estimates on explored trials but without reversing the patterns reported in the main text.

(B) We examined the locations of the choice-related hippocampal clusters that were observed at the group level (Figure 4A) in each subjects’ native space, by using the inverse mapping tools from the normalization protocol (see Supplementary Materials and Methods) to map the group-level clusters onto each subject’s scan. For each subject, voxels that demonstrated a main effect of choice (red) were located in the superior hippocampus (extending into the amygdala), whereas voxels demonstrating a condition x choice interaction (blue) were located in the inferior hippocampus.

**Figure S2: Interrogating the choice-related signals in the hippocampus**


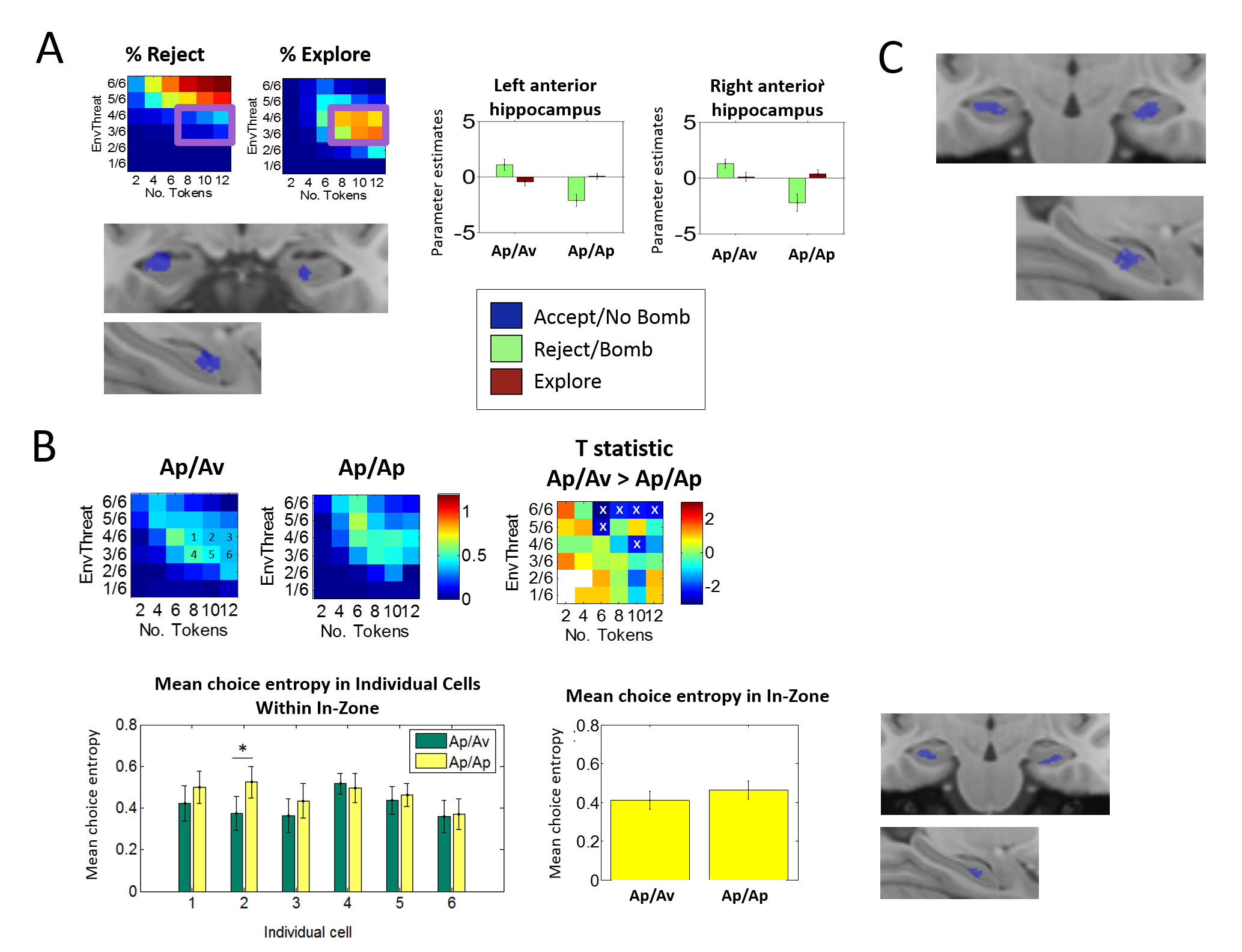


(A) In order to further examine the choice-related signals in the hippocampus, we considered if the hippocampus was supporting avoidance in the Ap/Av condition in more constrained parts of the task-space. Focusing restrictedly on a subset of the task space in which subjects rejected gambles only occasionally (cells outlined in purple; see ‘*restricted categorical choice’* model in Supplemental Materials and Methods for detail) again implicated the anterior CA1 in avoidance (left: peak at -26.8, 11.6, 9.9, 142 voxels, F=26.06, peak FWE p=0.006; right: peak at 20.2, 12, 6.8, 67 voxels, F=18.30, peak FWE p=0.058). This result was observed despite relative homogeneity in the task-related variables in the identified cells, which involved gambles with relatively low threat [p(Activated Bomb)]. Note that while the CA3/CA1 subfields are located on the superior/inferior sections of the hippocampus in more posterior sections of the hippocampus (including the coronal slice in Figure 4A), the clusters shown here are entirely in the CA1 subfield, because cells in this anterior-most portion of the hippocampus all belong to the CA1 subfield (see Wisse et al, 2012 for more detail).

(B) We considered the possibility that between-condition differences in inferior anterior hippocampal engagement (Figure 4A) might be confounded by greater engagement of forward simulation processes (which are thought to be hippocampal; see Redish, 2016, for review). Such between-condition differences in simulation processes might arise as a result of the greater stakes involved in the Ap/Av condition relative to the Ap/Ap. We calculated subjects’ choice entropy (i.e. variance in choosing between accept/reject/explore, or bomb/no bomb/explore) when facing each gamble as an index of subjective uncertainty, in order to assess whether differences in choice complexity may have biased our neural results. Specifically, we examined whether subjective uncertainty (i.e. average choice entropy) was higher in the Ap/Av compared to the Ap/Ap condition, for the subsets of trials in which participants rejected gambles only occasionally. Choice entropy was calculated separately for each cell of the 6x6 task space, in each of the Ap/Av and Ap/Ap conditions:

$$\sum_{c=1}^{3} -p(c) \cdot log(p(c))$$

where *p(c)* relates to the observed probability that the subject would choose that choice option when faced with that specific gamble (i.e. the specific individual cell of the 6x6) in the given experimental condition. Mean choice entropy across the 6x6 task space in both the Ap/Av and Ap/Ap conditions are shown in the top-left and top-middle of (B), with the t statistic for cell-wise Ap/Av > Ap/Ap comparisons shown on the top-right of (B) (white crosses indicate p <0.05, no adjustment made for multiple comparisons, all other p>0.1). If the between-condition differences in hippocampal engagement (i.e. in Figure 4A and Figure S2A) were the result of greater engagement of forward simulation processes in the Ap/Av condition, we would also expect to observe that subjective uncertainty (indexed by choice entropy) would be higher in the Ap/Av condition relative to the Ap/Ap. However, in the overall task space, significant differences in choice entropy are only observed in the direction of less subjective uncertainty in the Ap/Av condition relative to the Ap/Ap (i.e. t-statistic <0 for all cells where the cell-wise comparison is significant). We also examined if this was the case in the 6-cell in-zone, in which we had recapitulated the avoidance-related (condition x choice) effect in the anterior hippocampus (A). Contrary to what we might have expected to see had our hippocampal findings been confounded by subjective uncertainty, we did not observe higher choice entropy in the Ap/Av condition either considering the in-zone as a whole (bottom middle of B), or in any of these the 6 individual cells demarcated in the in-zone (bottom right of B). As shown in the (B), there is a significant between-condition difference in choice entropy in only one of these 6 cells (all p>0.1 except for cell no. 2, where t(19)=2.24, p=.037, numbered reference to cells in the overall task space are indicated in the top left panel of B), again indicating lower choice entropy in the Ap/Av condition rather than more. This is notable because the avoidance-related hippocampal result was observed even when we limited our analysis to this restricted portion of the task space (A). Finally, we ran an fMRI model that included (as regressors of no interest) choice entropy instead of the objective entropy quantities, this time consider choice over the entire 6x6 task space (i.e. not restricted to the in-zone demarcated in A). The observed results in the inferior hippocampus were robust to the inclusion choice entropy as a nuisance regressor, bottom right of (B) (right: peak at 26.3, 4, 9, F=11.22, peak FWE p=.029, Z=3.93, 87 voxels; left: peak at -25.7, 5, 12, F=10.71, peak FWE p=.041, Z=3.83, 97 voxels). As such, it seems unlikely that the neural results found in the inferior anterior hippocampus (Figure 4A; i.e. a condition x choice interaction, reflecting greater engagement for Ap/Av-reject > Ap/Ap -bomb) are reflective of greater engagement of simulation-related processes as related to subjective uncertainty, as opposed to avoidance per se.

(C) To further examine the possibility that the results in the inferior hippocampus may have related to subjective uncertainty, we ran a further fMRI model that included (as regressors of no interest) the RTs associated with each choice (separately for the Ap/Av and Ap/Ap conditions; RTs assumed to be an index of subjective trial-to-trial uncertainty). The observed results in the inferior hippocampus were robust to the inclusion of RT regressors.


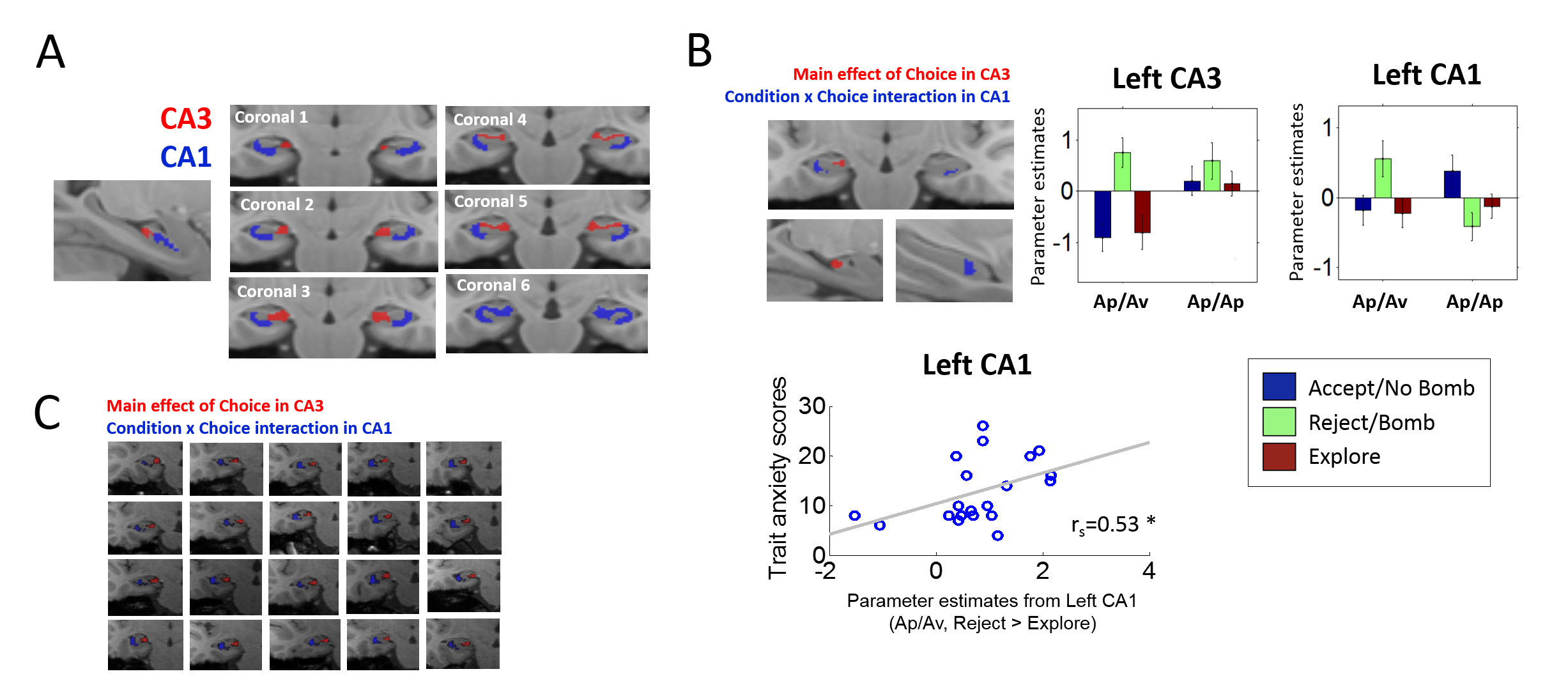
**Figure S3: Choice-related signals in the hippocampus**

(A) Estimated anatomical ROIs were created for the CA3 and CA1 subfields via manual segmentation, leaving out all voxels that could not be clearly classified (see Supplemental Materials and Methods for full detail). The sagittal slice shown is 28.6mm left from the midline, whereas the coronal slices shown start from the first slice (Coronal 1) where the uncus is visible (moving from the posterior to anterior of the brain), moving in an anterior direction in 1.3mm steps for 6 slices.

(B) We applied the estimated anatomical masks to the analysis of the categorical choice model results (i.e. as shown in Figure 3-4), and identified a main effect of choice and a condition by choice interaction in the left CA3 and bilateral CA1, respectively (see Table S3 for statistics and (C) for mapping in each subjects’ native space). The correlation with trait anxiety remained significant in the left CA1 (r_s_=0.53, *p*=0.017).

(C) Inverse mapping of these functional ROIs indicated that subfield affiliation of the group-level clusters were accurate in in each subjects’ native space.

We note that the spatial resolution of our data (functional: 3mm, structural: 1.5mm) does not allow for firm conclusions regarding the subfield affiliation of the different observed signals. As such, this analysis attempts to *estimate* the CA1 and CA3 signals rather than directly define them, and our attempts to map the inferior and superior hippocampal signals onto the underlying hippocampal subfields should be considered exploratory. Nevertheless, we include these results in order to highlight hippocampal subfield dynamics as a potentially productive focus for future work, since existing work on human hippocampal contributions to anxiety have typically omitted such a detailed anatomical focus.


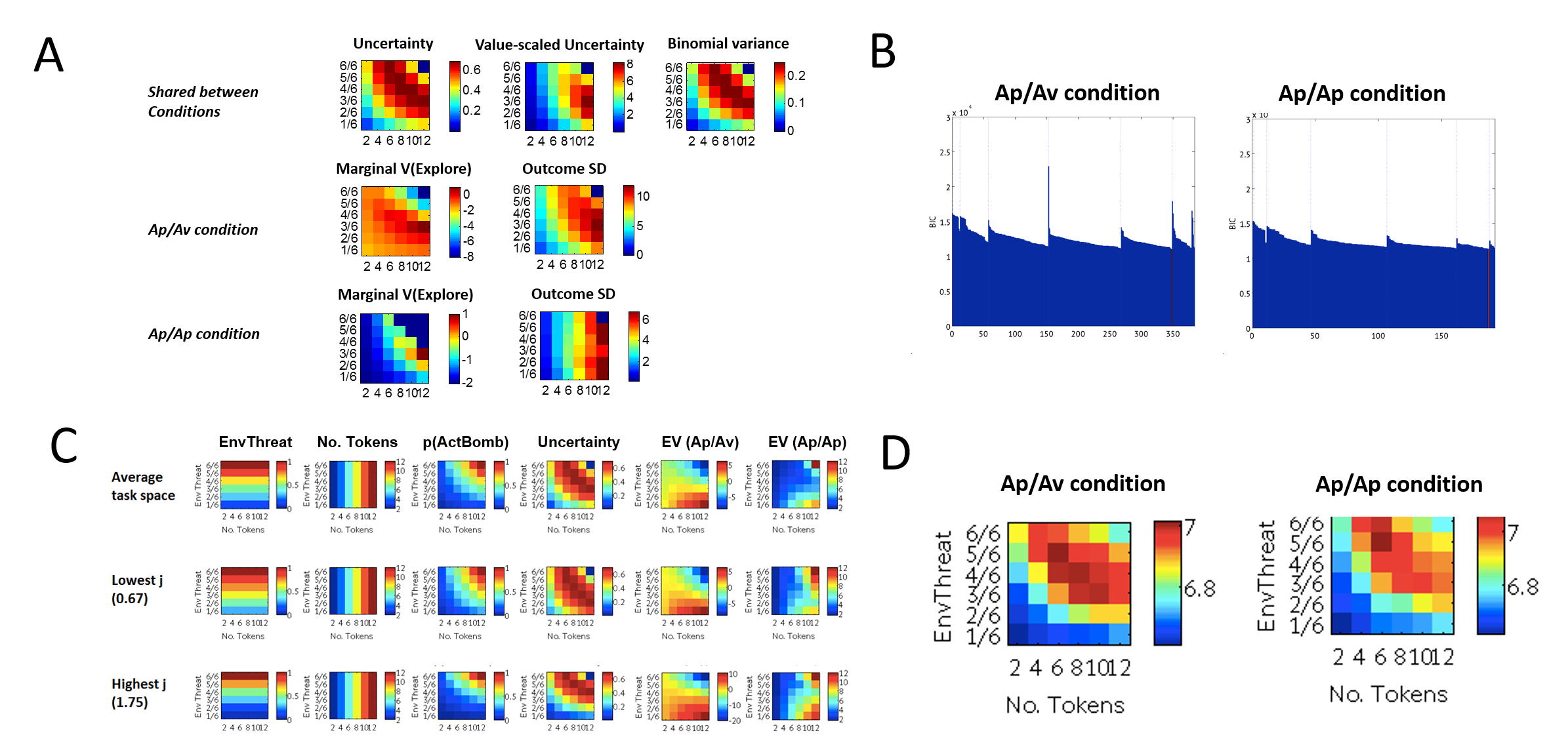
**Figure S4: Behavioural modelling**

(A) Several candidate quantities were considered as a driver for exploration (i.e. in modifying the exploration bonus across the task space) in the model space.

(B) BICs across the entire model space, organized by the number of parameters in each model. The red bar indicates the winning model, which and the lowest BIC.

(C) The winning model indicated that subjects slightly misestimated the environmental threat associated with each background colour. On average, the perceived task space experienced by subjects was limitedly distorted, relative to the actual probabilities. Note that vertical axis labels in (C) refer to the original Env Threat quantities, rather than the subjective quantities

(D Mean RTs (log-transformed) across the 6x6 task space in the Ap/Av and Ap/Ap condition

**Figure S5: Behaviour of excluded subjects**

(A) Example behaviour of subjects who were excluded because their choice behavior either indicated incorrect learning (top; i.e. choices do not reflect the correct environmental threat), or whose spontaneous choice would have led to poor power in our analysis of choice-related fMRI activity.

(B) Subjects who best learned the structure of the task were selected for the fMRI session, so as to maximise our chances of identifying choice-related activity in the brain. The excluded subjects did however show behaviour that was similar to that of the included subjects (Figure 2A)


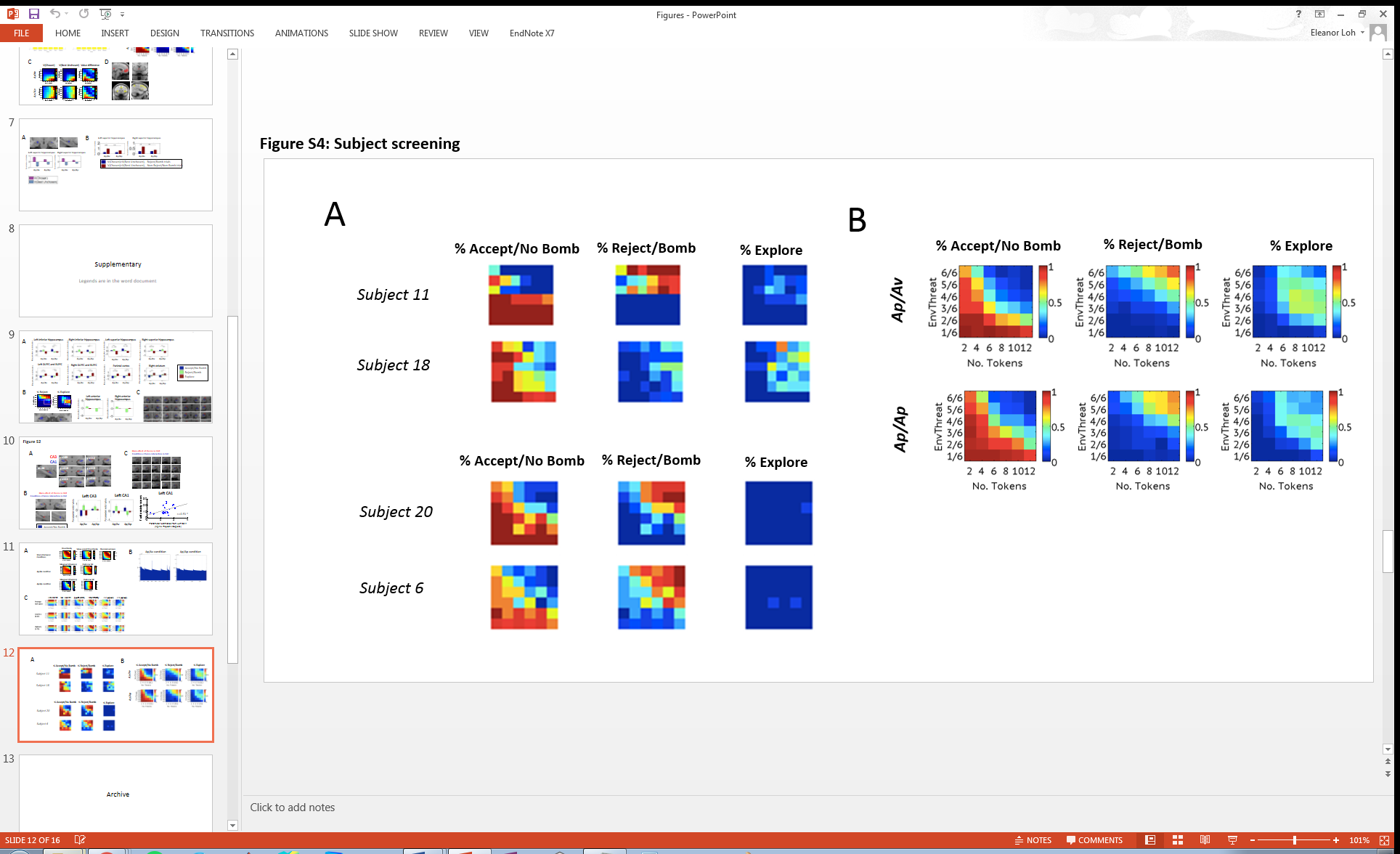


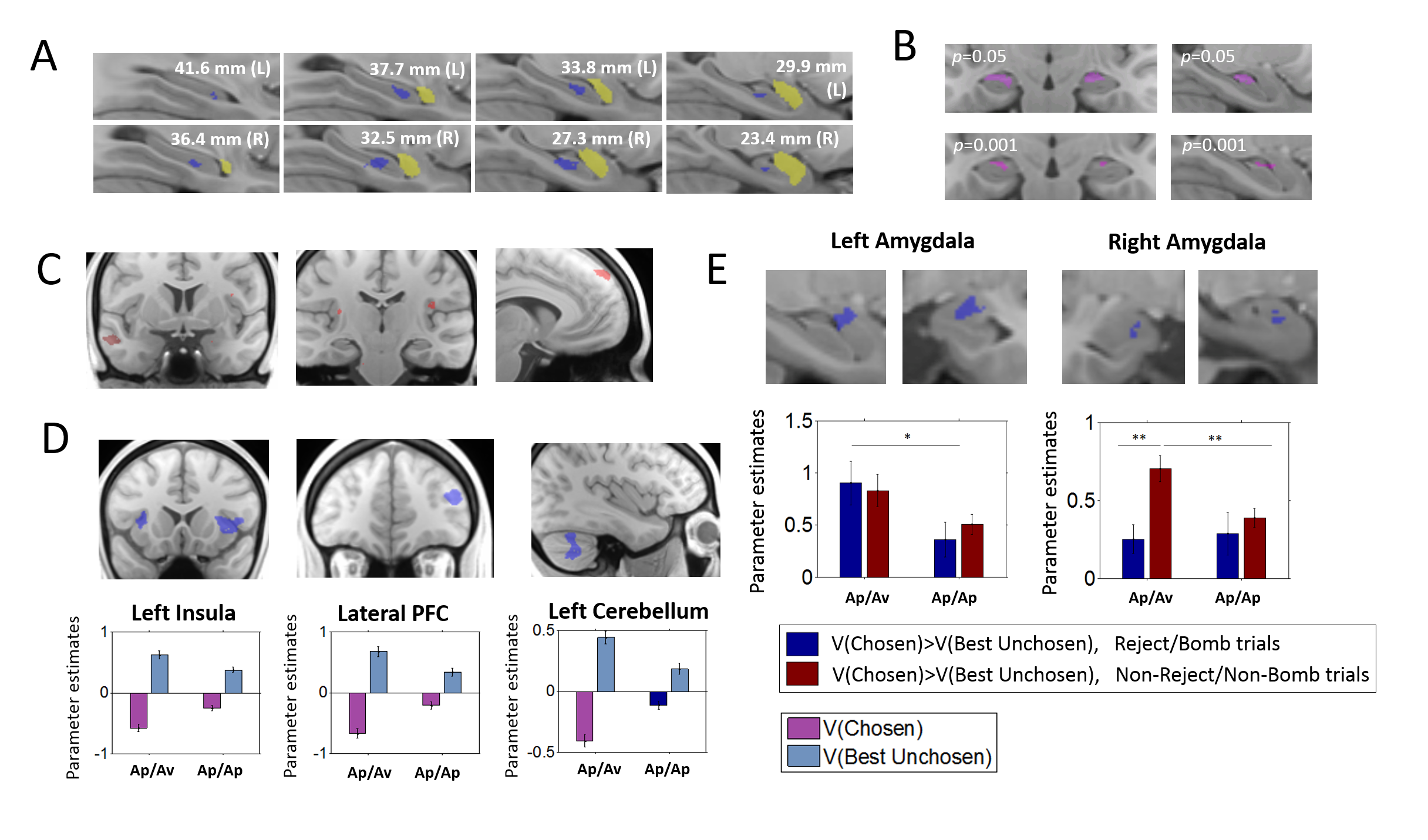
**Figure S6: Other choice and value signals in the brain**

(A) The inferior hippocampal ROI identified using the choice model (i.e. shown in Figure 4A) is presented here along with a structural image of the amygdala, to clarify the extent to which this functional ROI is spatially separated from the amygdala. The amygdala anatomical ROI was created by manually segmenting the amygdala on the group-level structural image, guided by an anatomical atlas (Mai, Paxinos, & Voss, 2008). All slices are shown with mm distance relative to the midline (L=left, R=right)

(B) Because two functionally distinct signals were observed in the hippocampus (see Figure 4A), we mutually masked the main effect of choice and condition x choice interaction contrast when examining the choice-related signals in the hippocampus. This is common practice within SPM, and aims to allow for the separate identification of voxels that show the main effect of choice or condition x choice pattern (see Materials and Methods for more detail). For completeness, however, a conjunction of these two contrasts is shown in this figure. The contrasts were mutually masked at the very lenient threshold of *p*=0.05 uncorrected (top, anatomically masked by the hippocampus and amygdala). We further include (for completeness) the same conjunction at a threshold of *p*=0.001 uncorrected. The voxels shown here demonstrated a mix of signals relating to the main effect of choice and condition x choice interaction, which is intrinsically difficult to interpret.

(C) Although no other cluster was found to survive family-wise-error correction, clusters in the temporal cortex (left: 543 voxels, peak Z=4.34, right: 113 voxels, peak Z=3.73), insula (right: 249 voxels, peak Z=3.87, left: 53 voxels, peak Z=3.56), rostral PFC (431 voxels, peak Z=3.87) were also noted for the *condition x value type* contrast reported in Figure 6A, at *p*=0.001 uncorrected. These regions, while not surviving FWE-correction, tracked value similarly to the hippocampus (i.e. potentiated value signals in the Ap/Av condition, relative to the Ap/Ap).

(D) We also examined the negative interaction in the condition x value type fMRI model. This contrast revealed FWE-significant clusters in the bilateral insula (right: cluster FWE p=0.009, 1183 voxels, peak Z=4.78; left: cluster FWE p=0.081, 703 voxels, peak Z=4.32), right lateral prefrontal cortex (cluster FWE p=0.033, 889 voxels, peak Z=4.60) and left cerebellum (cluster FWE p=0.004, 1379 voxels, peak Z=4.37). These clusters showed value signals that positively tracked counterfactual values were potentiated in the Ap/Av condition relative to the Ap/Ap. This contrasts with the results in the hippocampus, where chosen values were tracked positively and also potentiated in the Ap/Av condition relative to the Ap/Ap.

(E) Although we have focused on the hippocampus, we noted that the superior hippocampal value signals observed in our task did also extend into the amygdala (Figure 6A). For completeness, we repeated the same analytical steps used to identify the hippocampal signals in an exploratory analysis used to examine the value signals in the amygdala. Using the voxel-based approach, we first examined the condition x value contrast in the *chosen and counterfactual value* fMRI model (used to identify the clusters shown in Figure 5D and 6A), and identified amygdala voxels that tracked value differently in the Ap/Av versus Ap/Ap condition (uncorrected threshold of *p*<.001, SVC for the bilateral amygdala). After identifying clusters in the left and right amygdala in this manner (right: peak at 22.2, 14, 1, 144 voxels, F=22.76, Z=4.20, peak FWE p=0.003; left: peak at 20.1, 24, 8, 12 voxels, F=15.86, Z=3.55, peak FWE p=0.034), we then extracted parameter estimates from these clusters, from the *choice x value difference* model that split value differences according to whether subjects had chosen ‘bomb’ or ‘reject’ on that trial. These analysis steps, which are identical to the analytical procedure applied to the hippocampus, revealed a similar pattern of value tracking as had been seen in the hippocampus, in the right amygdala but not the left (see Table S5 for full statistics). Note that the interaction in the right amygdala, while qualitatively similar to that seen in the hippocampus, similarly remained at the level of a statistical trend. These results highlight the role of the amygdala in tracking value signals together with the hippocampus, on trials in which subjects have omitted to decisively avoid potential threats in the Ap/Av condition.

**Supplemental Tables**

Note: All coordinates are in arbitrary group space (not MNI space). See Supplemental Materials and Methods (spatial normalization) for more detail.

**Table S1: Cluster statistics for Categorical-Choice model**

|  |  |  |  |  | **Peak coordinates (mm)** | | |
| --- | --- | --- | --- | --- | --- | --- | --- |
|  | **Cluster size (no. voxels)** | **Peak FWE p** | **F** | **Z** | **x** | **y** | **z** |
|  |  |  |  |  |  |  |  |
| **Main effect of Choice** |  |  |  |  |  |  |  |
|  |  |  |  |  |  |  |  |
| Right DLPFC and RLPFC | 21678 | 0.000 | 36.07 | 6.92 | 24.2 | 25.9 | 70.8 |
|  |  | 0.000 | 28.61 | 6.26 | 24.0 | 48.9 | 58.9 |
|  |  | 0.000 | 25.79 | 5.98 | 23.2 | 33.9 | 74.8 |
| Parietal cortex | 27207 | 0.000 | 35.39 | 6.87 | 3.8 | -44.2 | 59.6 |
|  |  | 0.001 | 24.01 | 5.78 | 4.8 | -35.2 | 57.7 |
|  |  | 0.001 | 23.79 | 5.76 | 8.8 | -45.2 | 52.6 |
| Cerebellum | 6114 | 0.000 | 26.35 | 6.04 | -34.5 | -30.4 | -10.2 |
|  |  | 0.017 | 18.84 | 5.15 | -43.5 | -22.5 | -10.2 |
|  |  | 0.063 | 16.75 | 4.86 | -41.5 | -33.4 | -20.2 |
| Cerebellum | 2169 | 0.006 | 20.40 | 5.36 | 4.8 | -59.1 | 0.6 |
|  |  | 0.313 | 14.04 | 4.44 | 17.9 | -62.9 | -2.5 |
|  |  | 0.859 | 11.50 | 3.98 | -3.2 | -55.1 | 0.6 |
| Left RLPFC | 1681 | 0.011 | 19.54 | 5.24 | -26.4 | 79.5 | 38.1 |
|  |  | 0.651 | 12.45 | 4.16 | -21.3 | 78.6 | 46.1 |
|  |  | 0.716 | 12.18 | 4.11 | -36.3 | 70.4 | 40.1 |
| Left DLPFC | 7772 | 0.013 | 19.30 | 5.21 | -23.0 | 52.5 | 62.0 |
|  |  | 0.040 | 17.47 | 4.96 | -17.7 | 18.5 | 87.9 |
|  |  | 0.065 | 16.70 | 4.85 | -23.0 | 48.5 | 69.0 |
| Cerebellum | 7003 | 0.035 | 17.69 | 4.99 | 32.5 | -24.8 | -11.4 |
|  |  | 0.048 | 17.18 | 4.92 | 36.5 | -32.7 | -15.4 |
|  |  | 0.066 | 16.69 | 4.85 | 41.7 | -46.7 | 0.5 |
| Right striatum | 1762 | 0.036 | 17.63 | 4.99 | 19.2 | 24.9 | 49.8 |
|  |  | 0.256 | 14.41 | 4.50 | 19.1 | 33.9 | 43.8 |
|  |  | 0.263 | 14.35 | 4.49 | 18.3 | 10.9 | 50.8 |
| Left RLPFC | 1523 | 0.047 | 17.23 | 4.93 | -45.2 | 62.4 | 31.1 |
|  |  | 0.050 | 17.11 | 4.91 | -33.3 | 64.5 | 24.1 |
|  |  | 0.251 | 14.44 | 4.50 | -41.3 | 69.4 | 26.1 |
| Occipital cortex | 3678 | 0.048 | 17.20 | 4.92 | -11.0 | -73.3 | 20.6 |
|  |  | 0.100 | 16.00 | 4.75 | -21.1 | -63.4 | 15.7 |
|  |  | 0.267 | 14.33 | 4.49 | -18.0 | -68.3 | 22.6 |
| Right insula | 653 | 0.067 | 16.64 | 4.84 | 54.1 | 22.3 | 24.7 |
|  |  | 0.977 | 10.48 | 3.78 | 54.2 | 17.3 | 34.7 |
| Left hippocampus | 197 | 0.000 | 19.10 | 5.19 | -21.7 | 4.6 | 14.8 |
|  |  | 0.039 | 10.74 | 3.84 | -25.8 | 8.6 | 13.9 |
| Right hippocampus | 75 | 0.001 | 16.32 | 4.80 | 24.2 | 7.1 | 14.7 |
|  |  |  |  |  |  |  |  |
|  |  |  |  |  |  |  |  |
| **Condition x Choice** |  |  |  |  |  |  |  |
|  |  |  |  |  |  |  |  |
| Left hippocampus | 320 | 0.005 | 13.68 | 4.38 | -21.7 | 4.7 | 11.8 |
| Right hippocampus | 395 | 0.013 | 12.33 | 4.14 | 22.2 | 6.1 | 8.7 |
|  |  | 0.078 | 9.74 | 3.63 | 28.3 | 3.1 | 9.7 |
|  |  | 0.272 | 7.82 | 3.19 | 32.2 | 6.2 | 8.7 |
|  |  |  |  |  |  |  |  |

**Table S2: Statistics for choice-related effects in the Choice-only model**

| **Region of interest** | **Main effect of Condition** | **Main effect of Choice** | **Condition x Choice** | **Simple effects (interaction significant only)** |
| --- | --- | --- | --- | --- |
|  |  |  |  |  |
| Left inferior hippocampus | *nsf* | F(2,38)=12.2, p<0.001 | F(2,38)=8.91, p<0.001 | Ap/Av, Reject vs Explore t(19)= 4.41, p<0.001 Ap/Av, Accept vs Explore t(19)= 3.04, p= 0.007 Ap/Ap, NoBomb vs Bomb t(19)= 4.07, p<0.001 Ap/Ap, NoBomb vs Explore t(19)=3.97,p<0.001 |
|  |  |  |  |  |
| Right inferior hippocampus | *nsf* | F(2,38)=5.57, p=0.008 | F(2,38)=10.5, p<0.001 | Ap/Av, Reject vs Explore t(19)= 4.17, p<0.001 Ap/Av, Accept vs Reject t(19)= 3.39, p= 0.003 Ap/Ap, NoBomb vs Bomb t(19)= 3.92, p<0.001 Ap/Ap, NoBomb vs Explore t(19)=2.76,p=0.012 |
|  |  |  |  |  |
| Left superior hi  ppocampus | *nsf* | F(2,38)=32.5, p<0.001 | F(2,38)=7.94, p=0.001 | Explore, Ap/Av vs Ap/Ap: t(19)= 2.19, p= 0.041 Ap/Av, Reject vs Explore t(19)= 7.74, p<0.01 Ap/Av, Accept vs Reject t(19)= 2.4, p= 0.027 Ap/Av, Accept vs Explore t(19)= 5.76, p<0.01 Ap/Ap, Bomb vs Explore t(19)= 3.01, p= 0.007 Ap/Ap, NoBomb vs Bomb t(19)= 2.76, p= 0.012 Ap/Ap, NoBomb vs Explore t(19)=5.67,p= 0.01 |
|  |  |  |  |  |
| Right superior hippocampus | *nsf* | F(2,38)=19.5, p<0.001 | *nsf* |  |
|  |  |  |  |  |
| Right striatum | *nsf* | F(2,38)=11, p<0.001 | *nsf* |  |
|  |  |  |  |  |
| Left DLPFC and RLPFC | *nsf* | F(2,38)=19.5, p<0.001 | *nsf* |  |
|  |  |  |  |  |
| Right DLPFC and RLPFC | *nsf* | F(2,38)=40.9, p<0.001 | *nsf* |  |
|  |  |  |  |  |
| Parietal cortex | *nsf* | F(2,38)=31, p<0.001 | F(2,38)=3.87, p=0.0295 | Ap/Av, Reject vs Explore t(19)= 4.76, p<0.001 Ap/Av, Accept vs Reject t(19)= 2.64, p= 0.016 Ap/Av, Accept vs Explore t(19)= 5.93, p<0.001 Ap/Ap, Bomb vs Explore t(19)= 2.67, p= 0.015 Ap/Ap, NoBomb vs Bomb t(19)= 5.78, p<0.001 Ap/Ap, NoBomb vs Explore t(19)=6.75,p<0.001 |

**Table S3: Choice effects in CA3 and CA1**

|  |  |  |  |  | **Peak coordinates (mm)** | | |
| --- | --- | --- | --- | --- | --- | --- | --- |
|  | **Cluster size (no. voxels)** | **Peak FWE p** | **F** | **Z** | **x** | **y** | **z** |
|  |  |  |  |  |  |  |  |
| **Main effect of Choice** | |  |  |  |  |  |  |
|  | |  |  |  |  |  |  |
| Left CA3 | 47 | 0.013 | 12.34 | 4.14 | -19.7 | 5.7 | 11.8 |
|  |  |  |  |  |  |  |  |
| **Condition x Choice** |  |  |  |  |  |  |  |
|  |  |  |  |  |  |  |  |
| Right CA1 | 65 | 0.017 | 11.90 | 4.06 | 22.2 | 7.1 | 9.7 |
| Left CA1 | 82 | 0.033 | 10.98 | 3.88 | -23.8 | 6.6 | 11.9 |
|  |  | 0.077 | 9.75 | 3.63 | -27.8 | 5.6 | 10.9 |
|  |  | 0.129 | 8.99 | 3.46 | -24.8 | 5.6 | 7.9 |
|  |  |  |  |  |  |  |  |

**Table S4: Cluster statistics for Value model**

|  |  |  |  |  | **Peak coordinates (mm)** | | |
| --- | --- | --- | --- | --- | --- | --- | --- |
|  | **Cluster size (no. voxels)** | **Peak FWE p** | **T** | **Z** | **x** | **y** | **z** |
|  |  |  |  |  |  |  |  |
| **Main effect of Condition** |  |  |  |  |  |  |  |
|  |  |  |  |  |  |  |  |
| Parietal cortex (Ap/Ap>Ap/Av) | 5851 | 0.000 | 55.69 | 6.10 | -38.2 | -41.7 | 70.8 |
|  |  | 0.130 | 28.64 | 4.66 | -45.3 | -25.7 | 72.8 |
|  |  | 0.543 | 22.85 | 4.21 | -51.3 | -29.8 | 61.8 |
| Left RLPFC (Ap/Ap>Ap/Av) | 2324 | 0.036 | 33.31 | 4.97 | -37.3 | 67.4 | 38.1 |
|  |  | 1.000 | 13.44 | 3.27 | -28.3 | 73.5 | 39.1 |
|  |  | 1.000 | 13.22 | 3.24 | -43.2 | 57.4 | 34.1 |
| Left middle frontal gyrus (Ap/Ap>Ap/Av) | 1551 | 0.041 | 32.85 | 4.94 | -36.9 | 40.4 | 57.0 |
|  |  | 0.128 | 28.69 | 4.66 | -44.9 | 40.3 | 58.0 |
|  |  | 0.990 | 17.18 | 3.69 | -45.8 | 26.3 | 63.0 |
| Posterior cingulate (Ap/Ap>Ap/Av) | 1025 | 0.055 | 31.75 | 4.87 | 0.5 | -12.2 | 48.7 |
|  |  | 0.204 | 26.95 | 4.53 | -3.6 | -3.3 | 50.8 |
|  |  | 0.951 | 18.49 | 3.82 | 8.6 | -17.2 | 46.7 |
|  |  |  |  |  |  |  |  |
| **V(Chosen) > V(Best Unchosen)** | |  |  |  |  |  |  |
|  |  |  |  |  |  |  |  |
| VMPFC, bilateral hippocampus, bilateral temporal cortex | 261224 | 0.000 | 10.30 | 7.71 | -1.2 | 56.8 | 27.9 |
|  |  | 0.000 | 10.11 | 7.62 | -21.7 | 5.7 | 12.9 |
|  |  | 0.000 | 9.79 | 7.47 | -50.9 | 19.4 | 8.0 |
| Cerebellum | 1729 | 0.009 | 5.99 | 5.26 | 23.7 | -52.9 | -8.4 |
|  |  | 0.593 | 4.49 | 4.14 | 20.8 | -55.9 | -15.4 |
|  |  | 0.594 | 4.49 | 4.13 | 35.7 | -48.7 | -11.5 |
| Parietal cortex | 1965 | 0.016 | 5.81 | 5.13 | -11.3 | -17.5 | 92.8 |
|  |  | 0.579 | 4.50 | 4.15 | -19.4 | -14.5 | 67.8 |
|  |  | 0.632 | 4.45 | 4.11 | -19.3 | -21.5 | 90.8 |
|  |  |  |  |  |  |  |  |
| **V(Best Unchosen) > V(Chosen)** | |  |  |  |  |  |  |
|  |  |  |  |  |  |  |  |
| Right insula, Supplemental motor area | 47226 | 0.000 | 12.01 | 65535.00 | 31.0 | 41.1 | 31.8 |
|  |  | 0.000 | 10.63 | 65535.00 | 6.1 | 33.8 | 67.9 |
|  |  | 0.000 | 9.37 | 7.26 | 5.0 | 47.7 | 66.9 |
| Left insula | 4195 | 0.000 | 11.46 | 65535.00 | -29.0 | 38.5 | 31.0 |
|  |  | 0.000 | 8.84 | 6.99 | -27.9 | 34.5 | 39.0 |
| Cerebellum | 7439 | 0.000 | 8.72 | 6.92 | -31.5 | -31.4 | -6.2 |
|  |  | 0.001 | 6.58 | 5.65 | -36.5 | -33.4 | -18.2 |
|  |  | 0.014 | 5.86 | 5.16 | -34.4 | -38.4 | -1.2 |
| Right Parietal cortex | 21942 | 0.000 | 8.24 | 6.66 | 35.7 | -31.9 | 59.6 |
|  |  | 0.000 | 8.23 | 6.65 | 29.9 | -48.0 | 48.6 |
|  |  | 0.000 | 8.16 | 6.62 | 47.7 | -29.9 | 64.6 |
| Left Parietal cortex | 13537 | 0.000 | 7.73 | 6.37 | -28.3 | -33.5 | 60.8 |
|  |  | 0.000 | 7.27 | 6.09 | -38.3 | -28.6 | 58.8 |
|  |  | 0.001 | 6.72 | 5.74 | -26.1 | -49.5 | 58.7 |
| Thalamus, striatum | 7517 | 0.000 | 7.04 | 5.94 | 10.3 | 7.9 | 31.8 |
|  |  | 0.003 | 6.29 | 5.46 | 6.4 | -7.1 | 21.7 |
|  |  | 0.010 | 5.94 | 5.22 | 9.4 | -1.1 | 36.7 |
| Cerebellum | 2130 | 0.000 | 6.90 | 5.86 | -8.3 | -50.2 | -8.3 |
|  |  | 0.961 | 4.01 | 3.75 | -13.2 | -55.2 | 0.7 |
| Left DLPFC | 8483 | 0.001 | 6.64 | 5.69 | -41.0 | 45.3 | 59.0 |
|  |  | 0.003 | 6.32 | 5.48 | -38.7 | 17.4 | 56.9 |
|  |  | 0.095 | 5.24 | 4.71 | -50.8 | 23.2 | 64.0 |
| Left Striatum | 1108 | 0.016 | 5.80 | 5.12 | -11.8 | 19.7 | 26.9 |
|  |  | 0.088 | 5.26 | 4.73 | -8.9 | 24.7 | 35.9 |
| Posterior Cingulate | 1100 | 0.024 | 5.69 | 5.04 | 5.4 | 3.8 | 52.8 |
|  |  | 0.078 | 5.30 | 4.76 | 0.5 | -10.2 | 48.7 |
|  |  | 0.217 | 4.94 | 4.49 | -3.6 | -2.3 | 51.8 |
| Occipital cortex, inferior temporal gyrus | 933 | 0.052 | 5.44 | 4.86 | -36.3 | -41.5 | 13.8 |
| Occipital cortex | 2343 | 0.182 | 5.00 | 4.54 | -28.1 | -66.4 | 16.7 |
|  |  | 0.387 | 4.70 | 4.30 | -26.0 | -73.4 | 27.6 |
|  |  | 0.999 | 3.69 | 3.48 | -20.0 | -70.3 | 14.6 |
| Cerebellum | 887 | 0.195 | 4.98 | 4.52 | 33.6 | -32.8 | -4.4 |
|  |  | 0.763 | 4.32 | 4.00 | 24.6 | -34.9 | -4.4 |
|  |  | 0.999 | 3.72 | 3.50 | 25.7 | -45.9 | 3.6 |
|  |  |  |  |  |  |  |  |
| **Condition x Value type, positive interaction** | | |  |  |  |  |  |
|  |  |  |  |  |  |  |  |
| Left hippocampus | 183 | 0.018 | 4.32 | 4.00 | 19.2 | 11.0 | 11.8 |
|  |  | 0.050 | 3.95 | 3.70 | 24.2 | 11.1 | 11.7 |
|  |  | 0.066 | 3.84 | 3.60 | 30.2 | 6.1 | 11.7 |
| Right hippocampus | 127 | 0.037 | 4.05 | 3.78 | -21.8 | 6.7 | 12.9 |
|  |  |  |  |  |  |  |  |
| **Condition x Value type, negative interaction** | | |  |  |  |  |  |
|  |  |  |  |  |  |  |  |
| Left hippocampus | 183 | 0.018 | 4.32 | 4.00 | 19.2 | 11.0 | 11.8 |
|  |  | 0.050 | 3.95 | 3.70 | 24.2 | 11.1 | 11.7 |
|  |  | 0.066 | 3.84 | 3.60 | 30.2 | 6.1 | 11.7 |
| Right hippocampus | 127 | 0.037 | 4.05 | 3.78 | -21.8 | 6.7 | 12.9 |
|  |  |  |  |  |  |  |  |
|  |  |  |  |  |  |  |  |

**Table S5: Amygdala value signals by condition and choice**

|  |  | |  | | |
| --- | --- | --- | --- | --- | --- |
|  | **Left** | | **Right** | | |
| Condition x Choice (2x2) ANOVA (df=1,19) |  | |  | | |
|  |  | |  | | |
| ME Condition | | F=7.91, p=0.011 * | | nsf |  |
| ME Choice | | nsf | | F=5.76, p=0.027 * |  |
| Condition x Choice | | nsf | | F=4.29, p=0.052 |  |
|  | |  | |  |  |
|  | |  | |  |  |
| Simple effects (df=19) | |  | |  |  |
|  | |  | |  |  |
| Ap/Av, Reject vs Others | | nsf | | t= 3.53, p= 0.002 ** |  |
| Ap/Ap, Bomb vs Others | | nsf | | nsf |  |
|  | |  | |  |  |
| Reject vs Bomb  (Ap/Av vs Ap/Ap) | | t= 2.47, p= 0.023 * | | nsf |  |
| Accept/Explore vs No Bomb/Explore  (Ap/Av vs Ap/Ap) | | t= 1.96, p= 0.065 | | t= 3.82, p= 0.001 ** |  |
|  |  | |  | | |

nsf: *p*>0.1

**Table S6: Fitted parameter values for the winning behavioural models**

Parameter values for each subject (n=20, indicated by row) are shown. The last two rows include mean values and standard errors for each parameter.

| ***Ap/Av condition*** | | | | | |  | ***Ap/Ap condition*** | | | | | |
| --- | --- | --- | --- | --- | --- | --- | --- | --- | --- | --- | --- | --- |
| **β** | **ε *** | **j** | **i** | **f** | **w** |  | **β** | **ε *** | **j** | **i** | **e** | **w** |
| 2.05 | 0.43 | 0.78 | -1.43 | -7.72 | 1.49 |  | 1.02 | 0.37 | 0.95 | -5.28 | -4.83 | 4.36 |
| 3.61 | 0.54 | 1.18 | -1.43 | -8.87 | 2.84 |  | 2.22 | 0.28 | 1.22 | -3.05 | -3.10 | 5.13 |
| 5.39 | 0.16 | 0.94 | -1.25 | -5.97 | 2.65 |  | 2.58 | 0.24 | 1.41 | -3.15 | -3.49 | 5.45 |
| 3.12 | 1.56 | 1.37 | -0.56 | -11.05 | 0.85 |  | 0.89 | 0.24 | 0.70 | -7.11 | -5.55 | 4.54 |
| 3.37 | 0.57 | 0.95 | -1.38 | -8.31 | 2.81 |  | 1.58 | 0.21 | 0.96 | -3.12 | -3.89 | 5.49 |
| 2.74 | 0.74 | 1.06 | -1.96 | -8.96 | 2.49 |  | 1.62 | 0.20 | 1.17 | -2.09 | -2.15 | 3.34 |
| 3.36 | 0.18 | 1.12 | -1.28 | -8.01 | 2.62 |  | 2.36 | 0.32 | 1.38 | -3.14 | -4.36 | 6.26 |
| 2.92 | 0.48 | 1.30 | -0.15 | -13.39 | 1.21 |  | 1.16 | 0.31 | 0.93 | -3.89 | -5.40 | 6.83 |
| 4.08 | 0.23 | 1.04 | -1.73 | -7.02 | 2.96 |  | 2.21 | 0.25 | 1.44 | -2.96 | -4.27 | 5.69 |
| 6.54 | 0.47 | 0.72 | -1.76 | -8.84 | 2.49 |  | 2.99 | 0.28 | 1.33 | -2.19 | -2.90 | 3.82 |
| 2.31 | 0.45 | 0.84 | -1.98 | -9.50 | 2.91 |  | 1.81 | 0.21 | 1.03 | -1.75 | -1.42 | 3.13 |
| 2.32 | 0.41 | 1.75 | -0.46 | -20.22 | 1.16 |  | 3.11 | 0.27 | 0.99 | -2.54 | -2.71 | 4.81 |
| 2.97 | 0.79 | 0.98 | -1.50 | -7.51 | 3.20 |  | 2.37 | 0.79 | 1.31 | -0.75 | -0.39 | 1.88 |
| 2.10 | 2.20 | 0.79 | -1.34 | -11.76 | 1.94 |  | 0.90 | 0.49 | 0.67 | -4.66 | -4.85 | 5.58 |
| 2.28 | 0.47 | 0.78 | -0.92 | -9.61 | 1.63 |  | 1.98 | 1.17 | 1.25 | -2.54 | -3.95 | 5.13 |
| 4.22 | 0.81 | 0.72 | -0.74 | -8.82 | 1.08 |  | 1.78 | 1.08 | 0.90 | -3.99 | -4.88 | 6.24 |
| 4.51 | 0.21 | 1.63 | 0.80 | -14.85 | -0.36 |  | 6.62 | 0.51 | 1.48 | 1.06 | -8.15 | 1.43 |
| 3.22 | 3.58 | 1.29 | -0.58 | -12.24 | 1.35 |  | 1.03 | 2.04 | 0.95 | -4.28 | -4.30 | 5.54 |
| 3.21 | 0.66 | 0.87 | -1.44 | -8.48 | 2.35 |  | 6.83 | 2.59 | 1.50 | 1.00 | -8.35 | 1.44 |
| 3.39 | 0.36 | 1.06 | -0.97 | -9.38 | 2.38 |  | 1.42 | 0.27 | 1.47 | -2.33 | -3.89 | 5.41 |
| 3.39 | 0.77 | 1.06 | -1.10 | -10.03 | 2.00 |  | 2.32 | 0.61 | 1.15 | -2.84 | -4.14 | 4.58 |
| 0.25 | 0.18 | 0.07 | 0.15 | 0.73 | 0.21 |  | 0.37 | 0.15 | 0.06 | 0.43 | 0.43 | 0.36 |

* value multiplied by 100

**Table S7: Mean and SE correlations of regressors across value fMRI models (Pearson’s r)**

**Approach/Avoidance Condition**

|  | **Reject, V(Chosen) > V(Best Unchosen)** |  | **Accept/Explore, V(Chosen) > V(Best Unchosen)** |
| --- | --- | --- | --- |
| **V(Chosen)** | 0.0032 (0.0062) |  | -0.2100 (0.0157) |
| **V(Best Unchosen)** | 0.4144 (0.0271) |  | 0.1018 (0.0154) |

**Approach/Approach Condition**

|  | **Bomb, V(Chosen) > V(Best Unchosen)** |  | **No Bomb/Explore, V(Chosen) > V(Best Unchosen)** |
| --- | --- | --- | --- |
| **V(Chosen)** | 0.4323 (0.0297) |  | 0.1509 (0.0181) |
| **V(Best Unchosen)** | 0.0089 (0.0310) |  | -0.3821 (0.0142) |

**Supplemental Materials and Methods**

**Experimental procedure**

Subjects completed three different versions of this task, over four experimental sessions. In the first session (Learning stage), subjects completed a reduced version of the task in which they passively observed the associated outcome (win or loss) associated with each trial, and so learned which background colours were associated with the different levels of environmental threat, as well as how the probability of encountering bomb changed with each different combination of environmental threat and number of tokens. During this stage, in-activated bombs (i.e. bombs that were not under the tokens) were also shown after each trial outcome, to allow subjects to learn about the environmental threat in addition to learning about the overall outcomes associated with each gamble. Subjects were also instructed that they should monitor the fixation cross and press a button every time it changed from black to white. In order to prevent subjects from adopting risk-averse strategies for the remainder of the game, subjects were informed (after this session) that their winnings from this training session (typically large accumulated losses) did not count towards their monetary rewards, Subjects completed 432 trials in this training session (12 repetitions of the 36 trial types). Full instructions are listed in Appendix 1.

At the end of this training session, subjects completed a two-alternative-forced-choice task in which they chose between two of the background colours at a time. Subjects who did not learn the pairing between background color and environmental threat, or who could not explain (using worked examples, i.e. with known hypothetical environmental threats and a given number of tokens) how to correctly combine environmental threat and number of tokens to estimate p(ActBomb) were excluded from further participation in the experiment. For example, subjects who expressed the opinion that only environmental threat mattered to the outcome, or who could not rank the background colours in terms of ascending environmental threat, were excluded.

In the second session, subjects completed 432 trials of the Ap/Av condition alone (Figure 1A, bottom left). In the third session, subjects completed 432 trials of the Ap/Ap task (Figure 1A, bottom right; note order of sessions was not counterbalanced across all subjects). Subjects were allowed to progress to the next and final stage of the study if their verbal report after the third session and performance in these two sessions indicated comprehension of the task structure. At this point, we excluded subjects who:

1) changed their ranking of the background colours in terms of ascending environmental threat after either of the Ap/Av or Ap/Ap task session (as this indicated a change of mind regarding the background colours from the training session); 2) indicated in either their rankings or in their behaviour that they had mixed up the order of the background colours with respect to the associated threat levels (see Figure S5A, top, for examples); 3) failed to explore entirely (see Figure S5A, bottom, for examples); 4) could not verbally explain how information from the environmental threat and number of activated tokens should be combined to estimate p(ActBomb), which had been explained to them before the start of the experiment.

Subjects were extensively questioned, and their individual choice plots (i.e. similar to Figure 2A) visually inspected for these excluding criteria. Such strict screening procedures were employed to ensure that subjects whose data were included in the final analysis had reasonably similar estimates of p(ActBomb) across the 6x6 task space, so as to ensure that the psychological variables that were controlled for in our fMRI model (Figure 1B; see later sections for more detail) were reasonably accurate. Data from these training sessions were not subject to any further analysis, though winnings from these stages were included in the money given to subjects at the end of the experiment.

In the fourth session (fMRI stage, performed the next day), we collected fMRI data while subjects completed 12 alternating blocks of the Ap/Av and Ap/Ap condition with the starting condition counterbalanced across subjects (Figure 1C). Subjects were told that the p(ActBomb) associated with each gamble was the same as in previous sessions, and that this last session was an opportunity for them to use what they had learned so far to maximise their winnings in the game.

**Behavioural modelling**

In order to calculate value signals that may underlie choice, we fit a family of decision models to subjects' choices, parameterizing deviations from optimal behavior. Models calculated values on each trial for each of the three possible actions (accept/choose no bomb, reject/choose bomb, explore), which were then translated into choice probabilities via a softmax rule. The potential sources of sub-optimality were identified by considering both errors in optimal calculation (e.g. mis-estimation of Env threat, systematic miscalculation of the p(ActBomb) after one explores and fails to see a bomb), as well as descriptive psychological tendencies that could interfere with optimal performance (e.g. loss aversion, a tendency for overvalue exploration in some circumstances, a tendency to act according to null information revealed during exploration, rather than integrating this information into a revised estimate of p(ActBomb)). We constructed a large model space so as to consider a wide variety of possibilities regarding the manipulated quantities that influenced behaviour in our task. The following free parameters were considered in constructing the full model space (all parameters were included in both conditions, except the *f* parameter, which was applied only to the Ap/Av condition):

| Parameter | Description | Equation block no.  (in main text) |
| --- | --- | --- |
| β | Inverse temperature parameter of the softmax function; Governs the stochasticity of choice as a function of value | Equation S1 (Supplemental) |
| ε | Describes the irreducible stochasticity in choice | Equation S1 (Supplemental) |
| *j* | Distortion to environmental threat: EnvThreat^j^ | Equations 3 and 4 |
| *m* | Distortion to *k* [posterior p(ActBomb \| no bomb seen during exploration)], i.e. *k* = *k^m^* ; Allows for inoptimal calculation of posterior probabilities | Equation 3 [$V\left( No See \right)$]  Equation 4  [V_Stage 2 Accept_, V_Stage 2 Reject_] |
| *i* | Bonus to V_Stage 2 Accept_ and V_Stage 2 No Bomb;_ indexes general tendency to accept gambles/choose no bomb after exploration reveals a lack of a bomb [rather than integrating the null information into the estimate of p(ActBomb)] | Equation 4 (V_Stage 2 Accept_) |
| *f* | Perceived magnitude of the fixed loss (objectively equals to -12 tokens) in the Ap/Av condition only | Equation 3  [V(Accept), V(No See)] |
| *e* | Exploration bonus added to V(Explore) equally across the entire task space; quantifies the subjective cost of exploration (objectively equals to -2 tokens, as in equation 4) | Equation 4 [V(Explore)] |
| *w* | Bonus to V(Explore), quantifying the impact that task-related variables (e.g. uncertainty) have on V(Explore). Thus, this describes an exploration bonus that *varies* across the 6x6 task space. | Equations 3 and 4 [V(Explore)] |

Different versions of the *w* parameter were included in the model space so as to allow us to examine which task-related variables drove exploration (by modulating the exploration bonus) across the 6x6 task space. All variables considered for the variable exploration bonus *w* are shown in Figure S4A, and are as follows:

| Parameter | Description |
| --- | --- |
| *u* | Uncertainty regarding p(ActBomb) |
| *v* | Value gained from exploration, calculated as:  *v = optimal V(Explore) - EV*  See equations 1 & 2 for EV, and equations 3 and 4 for V(Explore) |
| *o* | Value-scaled uncertainty, calculated as:  *o* = $H\left( p\left( \mathrm{ActBomb} \right) \right) \times n$ |
| *y* | Binomial variance in p(ActBomb), calculated as:  *y* = p(ActBomb) *×* [ 1- p(ActBomb) ] |
| *s* | Standard deviation of outcome (EV) |

Comparison of models with the different versions of this *w* parameter thus allowed us to identify task-related quantities that influenced the trial-to-trial likelihood of exploration *across* the 6x6 task space. The *u* and *y* parameters describe an exploration bonus that is sensitive to uncertainty regarding p(ActBomb) (quantified in two distinct ways), whereas the *v* parameter describes a magnification of the marginal value of exploration that allows this marginal value to exert a greater (or reduced) influence on choice valuation that would be optimally expected. The *o* parameter describes an exploration bonus that is sensitive both to the information to be gained from exploration (i.e. uncertainty) as well as the monetary payoffs for that information gain, whereas the *s* parameter describes exploration as a response to risk (defined as payoff variance, in line with the framework proposed by D’Acremont & Bossaerts, 2008).

Values for each choice were then used to predict the probability of accepting/choosing no bomb, rejecting/choosing bomb or exploring on each trial via a softmax function:

$p\left( \mathrm{Choice} \right)= \varepsilon+ \left( 1- 3 \times\varepsilon\right)\times\frac{e^{\beta\times V(Choice)}}{e^{\beta\times V(Accept)}+ e^{\beta\times V(Reject)}+ e^{\beta\times V(Explore)}}$ (S1)

where β is the inverse temperature parameter of the softmax function (that governs the stochasticity of choice as a function of value), and ε describes the irreducible stochasticity in choice. The ε parameter is integrated into the typical softmax function as a fixed probability of making each choice, and the original p(Choice) (as would be calculated without the ε parameter) is scaled to ensure that the sum of p(accept), p(reject) and p(explore) sums to 1. Choice probabilities in the Ap/Ap condition were calculated similarly, substituting the values for choosing no bomb and bomb for the V(Accept) and V(Reject).

Normative values can be calculated by letting *m*=1*, j*=1*, f*= -12*, e*=-2*,* and *w*=0*.* Because the models were not nestable, separate models were included for all possible combinations of all free parameters (with different combinations of the *w* parameter included in the model space as distinct models, and no one model having more than one *w* parameter), producing 384 individual models in the Ap/Av condition and 192 in the Ap/Ap condition (which omits the *f* parameter). All models were allowed to compete on even footing with each other. Parameter fitting was implemented (separately for the Ap/Av and Ap/Ap condition) using a hierarchical type II Bayesian (random effects) procedure that used maximum likelihood to fit simple parameterized distributions for higher-level statistics of the parameters (Guitart-Masip et al., 2012; Huys et al., 2011). Models were compared using the integrated Bayesian information criterion (iBIC), in which small iBIC values indicate a model that fits the data better after penalizing for the number of parameters (to prevent over-fitting; Huys et al., 2011). iBIC values for the entire model space are shown in Figure S4B, and iBICs for the winning and neighbouring models (i.e. omitting individual parameters separately) are shown in Figure 6A.

Fitted parameter values for all subjects are shown in Table S6. Although the β parameter values were significantly higher in the Ap/Av condition (t(19)=2.84, p=0.010), the β parameter values are not directly comparable between the Ap/Av and Ap/Ap conditions due to differences in the ranges of values in the two conditions (on average: ranging from -10.03 to 8.44 in the Ap/Av, and ranging from -2.29 to 12 in the Ap/Ap) that would, all else being equal, lead to lower estimates of β in the Ap/Av condition relative to the Ap/Ap. Although significant differences were also observed in the values for the i and w parameters (which both impact V(Explore); respectively, t(19)=3.86, p=0.001; t(19)= 6.34, p<.001; p>0.3 for remaining shared parameters), the inclusion of the e parameter (which also modifies the value of exploration) in the Ap/Ap condition but not the Ap/Av makes differences in the i and w parameters difficult to interpret.

**fMRI analysis**

***Data acquisition and preprocessing*** Data acquisition was performed on a 3T Trio Siemens scanner (Siemens Healthcare, Erlangen, Germany) operated with a 32-channel head coil. Functional data was acquired using a three-dimensional gradient-echo T2*-weighted echo-planar imaging (EPI) sequence covering the entire brain (TR=70.0ms, TE=30ms; slab angled at -30° in the anteroposterior axis, 48 slices per volume acquired in ascending order; spatial resolution=3x3x3 mm). The functional imaging sequence chosen was optimized for orbitofrontal cortex and amygdala, with specialized shim in the Z plane to additionally optimize signal in the hippocampus (Weiskopf, Hutton, Josephs, & Deichmann, 2006). Respiration and heart rate were recorded using a breathing belt and pulse oximeter, and used to correct for respiration- and heartbeat-related artefacts (Hutton et al., 2011). Each subject underwent 6 session of functional scanning (roughly 260 volumes per session, each session lasting ~14 min), with breaks between each session for subjects to rest. Individual field maps were also acquired using the standard manufacturer’s double echo gradient echo field map sequence (TE = 10.0 and 12.46 ms, TR=1020ms; matrix size= 64x64; 64 slices, spatial resolution=3 x 3 x 3 mm), to allow for distortion correction using the SPM Fieldmap toolbox (Hutton et al., 2002). Multiparameter images, including T1-weighted, proton density, and magnetization transfer contrasts (spatial resolution=1.3 x 1.3 x 1.3 mm for all), were acquired for structural information using 3D FLASH (fast low-angle shot) sequences, using established multi-parameter map protocols (Weiskopf & Helms, 2008). Preprocessing of the fMRI data included bias correction, realignment, unwarping (using individual fieldmaps) and smoothing with a 4 mm Gaussian kernel. Standard spatial normalization steps were omitted during preprocessing, in lieu of the specialized protocols (using Advanced Normalization Tools, see below) that were applied after to data from the first-level contrasts.

***Spatial normalization*** Advanced normalization Tools was used to implement spatial normalization (Avants, Tustison, Wu, Cook, & Gee, 2011), in place of standard SPM spatial normalization protocols. We opted to implement spatial normalization in this way as it maximised the accuracy of spatial normalization, which enabled us to examine signals in sub-regions of the hippocampus with greater confidence. Transformations between each participant’s native space and the group template were established using the grayscale information present in the template and individual scans. This procedure produced affine and 3D diffeomorphic vector field transformations mapping each individual subject to the group template space. These transformations were then used to bring the first-level statistical maps (first-level contrasts, obtained by running the first-level models on the un-normalized but otherwise preprocessed data) from each subject into the group template space (using ANTS: WarpImageMultiTransform), to allow for generation of the second-level statistical activation maps. The inverse of these transformations were used to map clusters from group space back to the native space of each individual participant, to check whether clusters in individual subjects’ native space matched the pattern of results in the group-level results. Because all analysis was conducted in the group space, all reported cluster coordinates are arbitrary. All clusters are shown superimposed against the group-level template, in the figures.

***Hippocampal subfield segmentation*** To isolate any potentially subfield-specific choice effects in a more anatomically restricted way, we constructed estimated anatomical masks for the CA3 and CA1 hippocampal subfields, and used these anatomical masks to isolate voxels that showed different choice-related effects (i.e. demonstrating a main effect of choice vs a condition x choice interaction, in the categorical choice fMRI model). We focused on the CA3 and CA1 subfields because these sub-regions are anatomically segregated in the anterior hippocampus in a manner that mimics the segregation of the functional ROIs observed (shown in Figure 4A; i.e. the CA3 and CA1 sub-regions of the hippocampus are predominantly in the superior and inferior regions at the coronal slice in which the functional clusters were identified). Hippocampal subfield ROIs (CA3 and CA1; Figure S3A) were created by manually tracing coronal slices from the group template, using software developed by Hugo Kuijf and based on MeVisLab (MeVis Medical Solutions AG, Bremen, Germany). The bilateral anterior hippocampus was segmented separately for each hemisphere, with the first coronal slice where the uncus can be clearly seen counted as the first slice belonging to the anterior hippocampus. Although not all subfield boundaries were clearly visible on the T1 template with a 1.5mm isotropic resolution (especially the boundary between CA3 and DG) an estimation of the CA3 and CA1 subfields was segmented according to the recently published protocol of (Wisse et al., 2012), and based on experience from segmenting high-resolution data, using detailed landmarks described in the Atlas of the Human Brain were as additional guideline. Based on Wisse et al., (2012) the delineation of CA3 starts 1.4 mm anterior to the point where the uncus separates from the hippocampus on coronal images. In the atlas of the human brain there is also almost no CA3 in the anterior hippocampal head (Mai et al., 2008). Therefore, we began segmenting the CA3 two slices anterior to the point where the uncus separates from the hippocampus. The border between the CA1 and CA3 subfield was formed by the lateral-most point of DG, by drawing a vertical line to the superior border of the hippocampus. We were not able to trace CA2, which was therefore counted towards CA3. Due to the limited resolution, parts of dentate gyrus (DG) might also be counted towards CA3 as well as CA1. The boundary between CA1 and CA3, however, is clearly defined in the protocol. Due to the limited spatial resolution of the data, the results should be considered an approximation of the CA1 and CA3 subfields, rather than a direct delineation.

fMRI models

Five different general linear models (GLMs) were constructed, to analyse data from several different perspectives: (1) *categorical choice*, (2) *Choice only* (Supplemental materials only), (3) *restricted categorical choice* (Supplemental materials only), (4) *chosen and counterfactual value*, (5) *choice x value difference.* All models focused on the 2000ms epoch during which the gambles were presented onscreen, and during which subjects additionally had to make their choices (i.e. period indicated by the blue bar in Figure 1C). Orthogonalization of parametric modulators was omitted in the design matrix for all models, so as to ensure that parameter estimates relating to the regressors and parametric modulators compete for variance (Andrade, Paradis, Rouquette, & Poline, 1999). All four models also included additional nuisance regressors to remove variance associated with errors (on trials in which no valid response was recorded within the 2000 ms epoch), motor responses (time-locked to button presses), the presentation of outcomes on each trial (parametrically modulated by the *amount* of money won on that trial), and the presentation of novel information during exploration (omitted on 50% of the trials to allow for de-correlation of the fMRI BOLD signal). All regressors were convolved with the HRF including time and dispersion derivatives. Additional covariates were included to capture residual artifacts related to movement (three rigid-body translations and three rotations from realignment), scanning session, heart rate and respiration.

*Categorical choice model*  This model aimed to identify brain regions mediating choice, after controlling for manipulated task-related variables that might be tracked at a psychological or neural level (Figure 1B; namely, environmental threat, number of activated tokens, p(ActBomb), uncertainty, EV). Choice was modelled as 6 regressors of interest conforming to a 2x3, Task x Choice ANOVA design (accept/choose no bomb, reject/choose bomb and explore in the Ap/Av versus Ap/Ap condition). Choice regressors were modelled as stick functions in the GLM and time-locked to the response point in each two-second trial epoch (indicated by blue bar in Figure 1C, left). To remove variance associated with the aforementioned task-related variables, we included these variables as parametric modulators (separately for the Ap/Av and Ap/Ap condition), time-locked to the onset of each trial (Figure 1C). First-level contrasts pertaining to choices in model were included in a 2x3 (Condition x Choice) second-level ANOVA. This allowed us to identify choice effects that were not confounded by the task-related quantities that varied systematically across the task space. Specifically, we could thus examine (a) activations relating to the decision to reject gambles/choose bomb, after controlling for the passive tracking of aversive conditions [i.e. p(ActBomb)], and (b) activations relating to the decision to explore, after controlling for the passive tracking of the conditions that crucially drive exploration (i.e. uncertainty; see section on behavioural modelling in main text for more detail). Because the main effect of choice and condition x choice contrasts revealed distinct but partially-overlapping clusters in the hippocampus, we followed standard procedures and mutually masked two contrasts against each other (at a threshold of p=.05 uncorrected) in SPM, in order to identify signals that cleanly showed signals of each kind. Even unmasked, however, the two clusters showed a marked superior-inferior difference as described in the main analysis. Due to high correlations in the task-related variables as well as ambiguity regarding their interpretation (given their mutual competition), these task-related variables were not subjected to in-depth analysis at the second level.

*Choice only model* The categorical choice model opted to control for the psychological variables that varied systematically across the task space, because omission of such controls would have resulted in the choice effects being confounded by other task-related variables that varied systematically across the task space. This approach had allowed us to identify regions that were implicated in choice, separately from neural regions whose activation varied systematically across the task space (e.g. allowing us to identify regions involved in the *decision* to reject, without implicating regions that were involved in passively tracking p(ActBomb), which is correlated with rejecting behaviour across the task space). To verify that the choice-related effects identified using the first two models were robust to the exclusion of the regressors for the manipulated task-related variables, however, we built an additional fMRI model that modelled choice alone, as 6 regressors of interest (2x3, Condition x Choice), without including any regressors for the psychological variables. We analysed activation using this model combined with the functional ROIs identified in the categorical choice model (ie. in Figure 3 and Figure 4 in main text). Parameter estimates were analysed using a 2x3 Condition x Choice ANOVA.

*Restricted categorical choice* This model was a variation on the *categorical choice* model that aimed to examine choice in a restricted set of gambles wherein explore and reject trade off (trials outlined in Figure S2A). By examining choice in this particular region of the task space, we were able to compare trials in which choice differed despite relative homogeneity in the task-related variables, and in so confirm that that the anterior hippocampus is involved in threat avoidance even when subjects choose to reject gambles with relatively low threat [p(ActBomb)]. We selected combinations of env threat and no. tokens for which for which reject/choosing bomb and explore traded off (outlined in Figure S2A), and examined neural responses that related to choice in this zone. We chose to focus on a region of the task space in which reject/choose bomb and explore traded off, because attempts to clarify the condition x choice interaction observed in the inferior hippocampus (i.e. Figure 4A) indicated that the effects here were driven by between-condition differences in reject/choose bomb vs explore choices, without implicating acceptance/choosing no bomb. Within this restricted region of the task space, p(ActBomb) ranged from 1/3 to 2/3, and the overall mean percentages of in-zone reject/choose bomb were 24.3% and 19.6% in the Ap/Av and Ap/Ap conditions respectively. This model included 4 choice regressors of interest (reject/choose bomb and explore in the Ap/Av and Ap/Ap condition, in the target zone *only*) and 4 choice regressors of no interest (accept/choose no bomb and reject/choose bomb in the Ap/Av and Ap/Ap conditions, *outside* the specified task zone shown in Figure S2A). 4 subjects who were entirely deterministic in rejecting/choosing bomb versus exploring in the entire in-zone were excluded from this particular analysis (final n=16). First-level contrasts pertaining to reject/choose bomb vs explore choice in the in zone *only* were included in a 2x2 (Condition x Choice) second-level ANOVA (i.e. in-Reject/in-Choose-Bomb, in-Explore, for the Ap/Av and Ap/Ap conditions).

*Chosen and counterfactual value model*  Aside from examining behavioural choice, we were also interested to look for reinforcement-learning signals that the brain might compute in trying to determine strategic choice, such as values relating to the chosen and best unchosen (i.e. competing, counterfactual) option on each (Boorman, Behrens, & Rushworth, 2011; Hayden, Pearson, & Platt, 2009; Hunt, Dolan, & Behrens, 2014). It would not have been feasible to include regressors for these reinforcement learning signals in the categorical choice models, due to the high number of variables therein (and their mutual competition), which would have made interpretation highly ambiguous. Therefore, we constructed value models that omitted regressors relating to the task-related quantities (that had been included in categorical choice models), but instead included only the value regressors. Values of the chosen and counterfactual (best unchosen) options (as calculated using our computational models; see main text for more detail) were included as parametric modulators that were time-locked to the onset of each trial (separately for the Ap/Av and Ap/Ap condition; Figure 5D). To identify signals that tracked value *differently* in the Ap/Av and Ap/Ap conditions, first-level contrasts pertaining to V(Chosen) and V(Best Unchosen) were included in a second-level 2x2 (Condition x Value type) ANOVA.

*Choice x value difference model* This model aimed to compare neutral tracking of value differences on reject vs non-reject trials, and was motivated by earlier results that indicated that (a) the hippocampus was involved in rejecting aversive gambles, and (b) hippocampal value difference signals was potentiated in the Ap/Av condition compare to the Ap/Ap. This model included the following regressors: (a) regressors that modelled the trial event alone (with stick regressors, time-locked to the trial onset), separately for the Ap/Av and Ap/Ap condition, (b) parametric modulators indicating the value difference [i.e. V(Chosen) > V(Best Unchosen)] on rejected trials/trials in which subjects chose bomb only, separately for the Ap/Av and Ap/Ap condition, and (c) parametric modulators indicating the value difference on *non-rejected* trials only (i.e. trials on which subjects opted to accept/choose no bomb or explore), separately for the Ap/Av and Ap/Ap condition. Using this model, we examined the value signals in the hippocampal ROIs that had shown a Condition x Value type interaction, in the first value analysis (i.e. using the Chosen and Counterfactual Value; Figure 6A).

**Supplemental References**

Andrade, A., Paradis, A. L., Rouquette, S., & Poline, J. B. (1999). Ambiguous results in functional neuroimaging data analysis due to covariate correlation. *NeuroImage*, *10*(4), 483–486. http://doi.org/10.1006/nimg.1999.0479

Avants, B. B., Tustison, N. J., Wu, J., Cook, P. A., & Gee, J. C. (2011). An open source multivariate framework for n-tissue segmentation with evaluation on public data. *Neuroinformatics*, *9*(4), 381–400. http://doi.org/10.1007/s12021-011-9109-y

Boorman, E. D., Behrens, T. E., & Rushworth, M. F. (2011). Counterfactual choice and learning in a neural network centered on human lateral frontopolar cortex. *PLoS Biology*, *9*(6), e1001093. http://doi.org/10.1371/journal.pbio.1001093

D’Acremont, M., & Bossaerts, P. (2008). Neurobiological studies of risk assessment: a comparison of expected utility and mean-variance approaches. *Cognitive, Affective & Behavioral Neuroscience*, *8*(4), 363–374. http://doi.org/10.3758/CABN.8.4.363

Guitart-Masip, M., Huys, Q. J. M., Fuentemilla, L., Dayan, P., Düzel, E., & Dolan, R. J. (2012). Go and no-go learning in reward and punishment: Interactions between affect and effect. *Neuroimage*, *62-334*(1), 154–166. http://doi.org/10.1016/j.neuroimage.2012.04.024

Hayden, B. Y., Pearson, J. M., & Platt, M. L. (2009). Fictive reward signals in the anterior cingulate cortex. *Science (New York, N.Y.)*, *324*(5929), 948–950. http://doi.org/10.1126/science.1168488

Hunt, L. T., Dolan, R. J., & Behrens, T. E. J. (2014). Hierarchical competitions subserving multi-attribute choice. *Nature Neuroscience*. http://doi.org/10.1038/nn.3836

Hutton, C., Bork, A., Josephs, O., Deichmann, R., Ashburner, J., & Turner, R. (2002). Image distortion correction in fMRI: A quantitative evaluation. *NeuroImage*, *16*(1), 217–240. http://doi.org/10.1006/nimg.2001.1054

Hutton, C., Josephs, O., Stadler, J., Featherstone, E., Reid, A., Speck, O., … Weiskopf, N. (2011). The impact of physiological noise correction on fMRI at 7 T. *NeuroImage*, *57*(1), 101–112. http://doi.org/10.1016/j.neuroimage.2011.04.018

Huys, Q. J. M., Cools, R., Gölzer, M., Friedel, E., Heinz, A., Dolan, R. J., & Dayan, P. (2011). Disentangling the Roles of Approach, Activation and Valence in Instrumental and Pavlovian Responding. *PLoS Computational Biology*, *7*(4), e1002028. http://doi.org/10.1371/journal.pcbi.1002028

Mai, J. K., Paxinos, G., & Voss, T. (2008). *Atlas of the human brain* (3. ed). Amsterdam: Elsevier, Acad. Press.

Redish, A. D. (2016). Vicarious trial and error. *Nature Reviews. Neuroscience*, *17*(3), 147–159. http://doi.org/10.1038/nrn.2015.30

Weiskopf, N., & Helms, G. (2008). Multi-parameter mapping of the human brain at 1mm resolution in less than 20 minutes. *Proceedings of 16th ISMRM, Toronto, Canada*, 16:2241.

Weiskopf, N., Hutton, C., Josephs, O., & Deichmann, R. (2006). Optimal EPI parameters for reduction of susceptibility-induced BOLD sensitivity losses: a whole-brain analysis at 3 T and 1.5 T. *NeuroImage*, *33*(2), 493–504. http://doi.org/10.1016/j.neuroimage.2006.07.029

Wisse, L. E. M., Gerritsen, L., Zwanenburg, J. J. M., Kuijf, H. J., Luijten, P. R., Biessels, G. J., & Geerlings, M. I. (2012). Subfields of the hippocampal formation at 7T MRI: In vivo volumetric assessment. *NeuroImage*, *61*(4), 1043–1049. http://doi.org/10.1016/j.neuroimage.2012.03.023

**Appendix 1: Task instructions**

**Stage 1**

In this experiment, you will be playing a decision-making game in which you can win and lose money. In the 1^st^ stage of the experiment, you will not be able to influence your winnings at all – the computer will decide when you win money, and when you will lose. Your task is to learn to predict when you will win or lose, so that you can use this information to maximize your winnings in the *next* stage of the game.

The computer decides whether you win or lose on each trial based on two pieces of information. First we will explain what these two pieces of information are, and how they relate to the computer’s decision. Then, we will explain what you actually have to do, in this game.

The computer decides whether to plan a ‘bomb’ in each trial, or not

On each trial, the computer will make you an ‘offer’, consisting of 12 tokens and a background colour.

This is a token


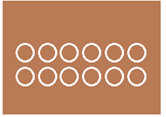


The first thing the computer will do is decide whether to place a ‘Bomb’ in the offer or not. It will not make this decision randomly – the probability of the computer placing a Bomb in the offer is determined by the background colour. There are 6 different background colours, and each one indicates a different probability of there being a bomb planted. For some colours, this probability will be high; but for others, it will be low. The relationship between each colour and the probability will stayed fixed throughout the entire experiment.


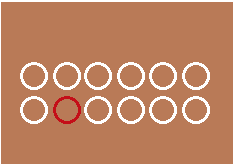


On this trial, the computer has paced a bomb here


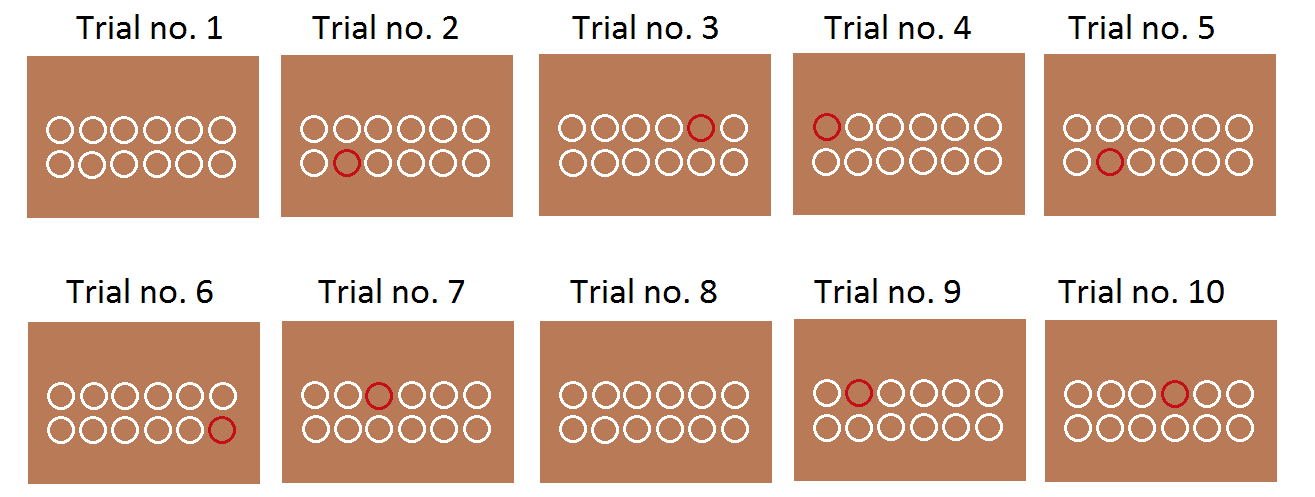
For example, say this brown background indicates an 80% likelihood of a bomb being present. This means that if you saw a trial with a brown background 10 times, the computer would have put a bomb in the offer in 8 out of the 10 times. Like this:

If there is a bomb in the offer, there will only be ONE bomb. The bomb will be randomly placed under any one of the 12 tokens.

***What happens if there is a bomb?*** Bombs will sometimes cause you to lose money – but only sometimes. Whether a bomb causes you to lose money or not depends on whether it is ‘activated’. The background colour tells you nothing about whether a bomb is likely to be activated or not. To find that out, you need to look at other details in the offer – we’ll explain that now.

The number of ‘activated’ tokens tells you how likely a bomb is to be ‘activated’

On each trial, the computer will also decide how many tokens to ‘activate. The number of activated tokens will range from 2 to 12. ‘Activated’ tokens are filled in white, like this:


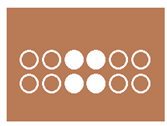


This offer consists of:

1. Brown background
2. 4 activated tokens out of 12 (coloured in white)

The number of activated tokens tells you two things. Firstly, it tells you how much money you will win on that trial, if the computer decides to give you money. Each activated token represents +10 p of money that you could win.


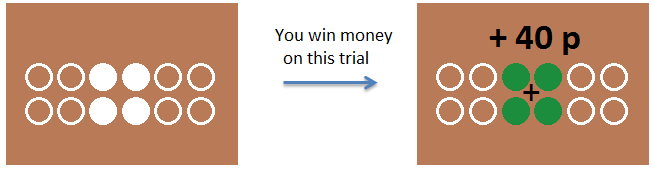


The number of activated tokens also tells you the likelihood of a bomb being ‘activated’, on trials where the computer has decided to plant a bomb. A bomb that is planted under one of the ***activated*** tokens (coloured in white) is ‘activated’, and will cause you to lose money. If the computer decides to plant a bomb, but it falls under one of the *inactivated* tokens, it will NOT cause you to lose money – on trials like this, you will still win.

On every trial, you will have to decide whether or not you think there is an activated bomb or not. It is only *activated* bombs that you should worry about - inactivated bombs will not cause you to lose money. To predict whether you will win or lose, you will need to pay attention to the background colour and the number of activated tokens.

***How exactly does the number of activated tokens affect my odds of losing?***

The likelihood of a bomb being ‘activated’ (if the computer has planted a bomb) is just a matter of probability:

*More activated tokens = Higher probability of there being an activated bomb*

*(given the same background colour)*

You can think of it as being similar to the odds of encountering a land-mine when walking across a dangerous plot of land. If there is a land-mine somewhere in the plot of land, your odds of setting it off would increase if you were to step on more places within the plot. If there IS a land-mine around, but you do NOT step on it, then it won’t go off, and you’ll be absolutely fine. And of course, if there are no land-mines at all in the plot of land (e.g. similar to Trial no. 1, in the picture on page 1), then it doesn’t matter how many places you step on – there is no bomb to set off, so you cannot possibly lose.


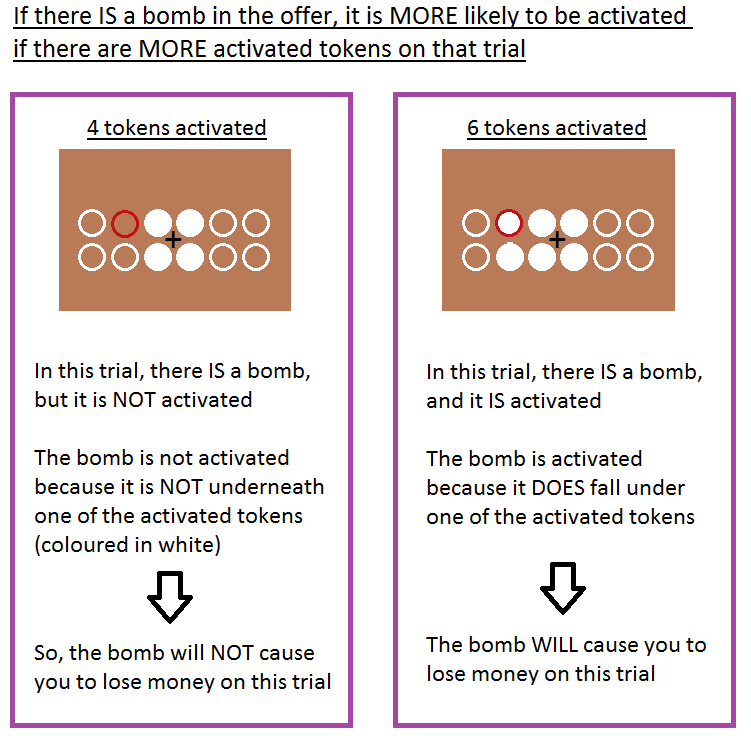


Stepping on more places in this plot of land is similar to having more activated tokens, in this game. The computer plants bombs on the different trials, with a probability indicated by the background colour. The background colour tells you about how ‘dangerous’ the trial is, generally – but even on dangerous trials, you won’t necessarily lose money. For any given background colour, increasing the number of activated tokens increases the odds that you will encounter an activated bomb, and lose money. Depending on how dangerous the trial is generally (as indicated by the background colour), more activated tokens may or may not mean that you are likely to encounter an activated bomb and lose money overall.

All in all, this means that with more activated tokens, the odds of losing go up. More activated tokens also means more money that you could potentially win, however. Thus there is an inherent trade-off between your potential winnings and the risk involved.

How do I play this game?

Now you know how the game works – how the computer decides whether you win or lose on each trial. Now we will show you how each trial will work.

On each trial, the computer will not show you the bombs straightaway. Each trial will go like this:


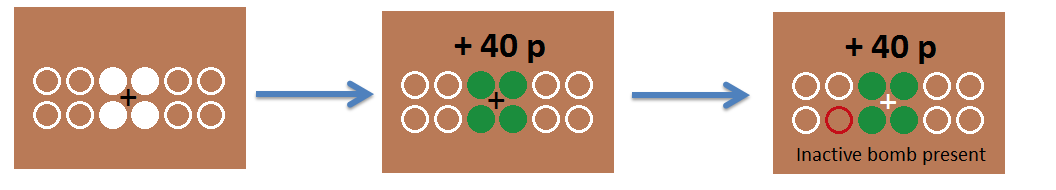


First, it will show you the offer itself, consisting of the background colour & number of activated tokens. Then it will show you how much money you won or lost, on that trial. Then, it will show you whether there were any inactivated bombs or not. For each trial, you should try to figure out whether you think there is likely to be an activated bomb or not. For trials where there is an activated bomb, you will lose – 120 p.

The inactive bombs do not have any effect on whether you win or lose money. We show you the inactive bombs because we want you to figure out, over the course of this session, how likely there is to be a bomb (activated or not) given each of the 6 different background colours. By the end of this session, you should be able to rank the colours in order of ‘best’ (least likely to have a bomb) to worst.

On trials where you lose money, we will not show you inactivated bombs, because you should already know that there was a bomb under one of the activated tokens.


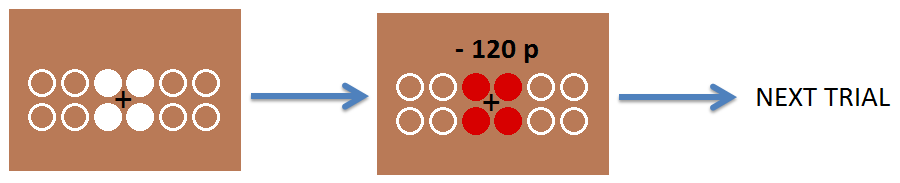


Note: If it isn’t clear to you how you would know whether there is a bomb (activated or not) on each trial, please ask the experimenter for more explanation!

Also: Look out for the white cross

While you are trying to figure out which colours are good and bad, you will also have to look out for the cross in the middle of the screen. Sometimes, this cross will turn white in colour – press the DOWN ARROW every time you see this cross turn white.


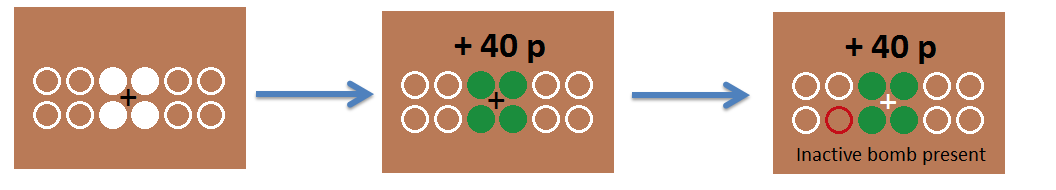


To summarize

Here is a summary of how the computer decides whether you win or lose on each trial:

1. The computer will decide whether to plant a bomb or not, as indicated by the background colour.
2. The number of activated tokens tells you how much money you could win on that trial, as well as how likely a bomb is likely to be activated (if there is a bomb there at all).
3. Activated bombs cause you to lose money (- 120p at a time), but inactivated bombs have no effect on whether you win or lose on that trial.

In this session, you will have to:

1. Look out for the white cross in the centre of the screen
2. Figure out how likely there is to be a bomb, given the 6 different background colours

By the end of this session, you should have a good idea how likely there is to be an *activated* bomb, given different combinations of background colour + number of activated tokens.

This information will be very important in helping you win money in the next stage of the experiment.

In the NEXT stage of the experiment, you will be able to avoid trials where you think there is likely to be an activated bomb. This is why it is important that you use this session to figure out when there is likely to be an activated bomb.

The extra money that we pay you at the end of this experiment will be proportional to the amount of money that you win on all the games in the experiment, so it’s important that you learn this game well, and win as much money on it as you can.

*This is a complicated game, and it’s important that you understand clearly how it works – you will lose a lot of money otherwise! Please ask the experimenter if anything is unclear about the game so far.*

**Stage 2**

By now, you should have a good idea of how likely there is to be an 'activated' bomb, given combination of background colour and number of tokens. By now, you should have a good idea of how likely there is to be an activated bomb, given combination of background colour and number of tokens. In this stage of the experiment, these same likelihoods apply. i.e. If a certain combination of background colour & number of activated tokens meant a good chance of there being an activated bomb in the previous stage of the experiment, it will indicate the same thing in this stage of the experiment. Thus, if a certain combination of background colour & number of activated tokens meant a good chance of there being an activated bomb in the previous stage of the experiment, it will indicate the same thing in this stage of the experiment.

In this stage of the experiment, however, you will perform a slightly different task. On each trial, the computer will make you an offer, consisting of a number of activated tokens and a coloured background. Like before, the background colour indicates the probability of there being a bomb in the offer. Just like in the previous stage, more activated tokens means greater potential winnings, but also a greater likelihood of encountering an activated bomb.

Note: The offers indicate both (a) the amount of money you can win as well as (b) how likely you are to win or lose. Please ask the experimenter if you’d like a reminder of how the game works!

When you see the offer, you should have a rough idea about whether there is likely to be an activated bomb or not - since you’ve learned this in the previous stage of the experiment. For each offer, you have the option of Accepting, Rejecting, or Exploring the offer. Make your choice by pressing the Left, Down or Right arrow respectively (with your RIGHT hand)

If you ACCEPT an offer with no activated bomb you will win money - 10p per activated. But if you Accept an offer WITH an activated bomb, you will LOSE money. Each time this happens, you will lose 120p. The computer will tell you whether you have won or lost money, on each trial.

You can also Reject offers, to avoid losing money. If you Reject the offer, you will not win or lose any money, no matter what outcome goes with the offer. Also, the computer will NOT tell you what outcome you would have gotten, if you had accepted the offer

If you always Reject offers, however, you will never win any money. Sometimes it’s better to Explore. f you choose to Explore, the computer will give you more information, before you choose to Reject or Accept the offer. The computer will reveal the status of 50% of the activated tokens offered, showing whether there are bombs or not, underneath these tokens. For each token, a green outline indicates NO bomb while a red outline indicates a bomb. After you learn the status of half of the activated tokens, you will then have to decide whether to Accept or Reject the offer.

Once again, you will receive the associated outcome if you Accept the offer, but you will not win or lose money (nor learn what the outcome would have been) if you choose to Reject the offer. The computer won’t tell you about inactivated bombs either, in all the rest of the experiment. They won’t have any effect at all on your winnings, throughout the experiment

The information gained by Exploring can help you decide if it is worth risking loss, to gain more money. This information is not free, however! Each Explore' choice will cost you 20p regardless of whether you decide to Accept or Reject the offer later on. If you use the Explorations well, you will end up winning more money overall, so it’s worth your while to try it out if you feel like you’d like more information before making your decision

You will have TWO seconds, for each response you have to make. If you do not respond in time, the trial will be stopped, and you will have to redo that trial again later in the game. Don’t worry if it seems like it’s going very quickly at first! You will get used to the pace and if you are too slow on a trial, you won’t lose, any money because of it. All that will happen is that you will have to do it later

Remember, you are playing for real money in this game - the amount of extra money we pay you at the end of this experiment will be proportional to your winnings in this game. Therefore, you should whatever you think would be the most likely to win you money on every single trial!

**Stage 3**

By now, you should have a good idea of how likely you are to encounter an activated bomb, given each type of background, as well as each combination of background colour and number of activated tokens

In this stage of the experiment, these same likelihoods apply. i.e. If a certain combination of background colour & number of activated tokens meant a good chance encountering an activated bomb in the previous stage of the experiment, it will indicate the same thing in this stage of the experiment

In this stage of the experiment, an activated bomb will NOT always lose you money. Your job in this stage of the experiment is to decide whether or not you think there is an activated bomb or not, on each trial. You will win money for correctly predicting whether there is an activated bomb on each trial

On each trial, the computer will show you the offer, consisting of a number of activated tokens and a coloured background. Press the Z key if you think there is NO activated bomb in the offer, or the X key if you think there IS an activated bomb in the offer. Use your LEFT HAND for this task. Press the Left arrow key if you think there is NO activated bomb in the offer, or the DOWN arrow if you think there IS an activated bomb in the offer. Use your RIGHT HAND for this task

For every trial where you correctly predict whether or not there is an activated bomb, you will win money. The amount of money you win is proportionate to the number of activated tokens (10p per token. For every trial where you are INCORRECT in your guess, you will NEITHER win NOR lose money. Unlike in the last stage of the experiment, you will NOT lose any money in this task.

You can also choose to Explore, if you'd like more information before making your guess. Press the C key with your left hand, if you'd like to Explore. If you choose to Explore, the computer will reveal the ''status'' of 50% of the activated tokens, showing whether there are bombs or not, underneath. A green outline indicates NO bomb, while a red outline indicates a bomb, underneath each token. After you learn the status of half of the activated tokens, you will then have to decide whether you think there is likely to be an activated bomb or not, on that trial.

The information gained by Exploring can help you decide which response to make. The information gained by Exploring can help you decide which response to make. But this information is not free - each ''Explore'' choice will cost you 20p, regardless of what choice you make after that

You will have TWO seconds, for each response you have to make. If you do not respond in time, the trial will be stopped, and you will have to redo that trial again later in the game.

In this stage, every single trial has equal influence on how much extra money you win on the task. The amount of extra money we pay you at the end of the experiment is proportionate to the total amount of money you win on these tasks. This means that every trial counts for real money and you should try your best to do whatever would be the most likely to win you money on every single trial
